# Genome-centric metagenomic insights into the role of Chloroflexi in anammox, activated sludge and methanogenic reactors

**DOI:** 10.1186/s12866-023-02765-5

**Published:** 2023-02-21

**Authors:** Patricia Bovio-Winkler, Leandro D. Guerrero, Leonardo Erijman, Pía Oyarzúa, María Eugenia Suárez-Ojeda, Angela Cabezas, Claudia Etchebehere

**Affiliations:** 1 Microbial Ecology Laboratory, Department of Microbial Biochemistry and Genomic, Biological Research Institute “Clemente Estable”, Avenida Italia 3318, CP: 11600 Montevideo, Uruguay; 2 Instituto de Investigaciones en Ingeniería Genética Y Biología Molecular “Dr Héctor N. Torres” (INGEBI-CONICET), Buenos Aires, Argentina; 3 GENOCOV Research Group, Department of Chemical, Biological and Environmental Engineering, Escola d’Enginyeria, Universitat Autònoma de Barcelona, 08193 Bellaterra, Spain; 4 Instituto Tecnológico Regional Centro Sur, Universidad Tecnológica, Francisco Antonio Maciel S/N, CP: 97000 Durazno, Uruguay

**Keywords:** Chloroflexi, Methanogenic reactors, Activated sludge, Anammox, Metagenome assembled genomes

## Abstract

**Background:**

The phylum Chloroflexi is highly abundant in a wide variety of wastewater treatment bioreactors. It has been suggested that they play relevant roles in these ecosystems, particularly in degrading carbon compounds and on structuring flocs or granules. Nevertheless, their function is not yet well understood as most species have not been isolated in axenic cultures. Here we used a metagenomic approach to investigate Chloroflexi diversity and their metabolic potential in three environmentally different bioreactors: a methanogenic full-scale reactor, a full-scale activated sludge reactor and a lab scale anammox reactor.

**Results:**

Differential coverage binning approach was used to assemble the genomes of 17 new Chloroflexi species, two of which are proposed as new Candidatus genus. In addition, we recovered the first representative genome belonging to the genus ‘Ca. Villigracilis’. Even though samples analyzed were collected from bioreactors operating under different environmental conditions, the assembled genomes share several metabolic features: anaerobic metabolism, fermentative pathways and several genes coding for hydrolytic enzymes. Interestingly, genome analysis from the anammox reactor indicated a putative role of Chloroflexi in nitrogen conversion. Genes related to adhesiveness and exopolysaccharides production were also detected. Complementing sequencing analysis, filamentous morphology was detected by Fluorescent in situ hybridization.

**Conclusion:**

Our results suggest that Chloroflexi participate in organic matter degradation, nitrogen removal and biofilm aggregation, playing different roles according to the environmental conditions.

**Supplementary Information:**

The online version contains supplementary material available at 10.1186/s12866-023-02765-5.

## Background

The phylum Chloroflexi comprises an ecologically and physiologically diverse group of bacteria, which have been detected in high abundance in methanogenic [1, 2], anammox [3–5] and activated sludge reactors [6, 7]. Despite of being highly abundant in wastewater treatment systems (WWTS), the basic ecophysiology of this group is still largely unknown because only few pure cultures have been investigated to date [8–13]. Based on the physiology of isolates, assembled genomes from metagenomes and in situ characterization (e.g., using microautoradiography combined with FISH), it has been proposed that their role is mainly related to the hydrolysis of complex organic matter, fermentation of carbohydrates and proteins, and degradation of debris from lysed bacterial cells [1, 7, 14–18].

In methanogenic reactors it has been hypothesized that the prevalence of this group is based on a syntrophic association with hydrogenotrophic methanogenic Archaea. This idea is supported by the fact that all Chloroflexi species isolated from methanogenic reactors required co-cultivation with methanogenic archaea for efficient growth [8, 10, 19, 20], the co-localization with filamentous Archaea [17], and the positive correlation between Chloroflexi and methanogens in anaerobic reactors [2].

In Anammox reactors, ammonium oxidation and nitrite reduction are coupled to form nitrogen gas under anoxic conditions [21]. In these reactors, the role of Chloroflexi has been associated to the scavenging of organic matter derived from anammox bacterial cell debris [3], or the utilization of soluble microbial products (SMP) and/or extracellular polymeric substances (EPS) produced by autotrophs [22]. This is supported by the high abundance of Chloroflexi in anammox reactors fed with synthetic wastewater containing NH4+ as the sole electron donor without addition of organic carbon compounds [3, 23–25]. Recent metagenomic-based works also suggested that members of Chloroflexi could facilitate a nitrite loop with anammox bacteria or support complete denitrification due to the expression of the nitric oxide reductase gene (norZ) and nitrite reductase genes (nirK, nirS). Thus, Chloroflexi would enhance the overall nitrogen removal performance in anammox bioreactors [26, 27]. In addition, some Chloroflexi members encode the function of biosynthesizing sticky macromolecular exopolysaccharide for anammox consortium aggregation [27].

On the other hand, it has been widely speculated that Chloroflexi members might play an important role during sludge granulation or flocculation processes. Evidence for this is their growth as filaments and the fact that some members showed cellular adhesiveness. This would enable Chloroflexi to form a backbone for small sludge particles and granules, in anammox, anaerobic and aerobic reactors [22, 28, 29]. Due to their filamentous growth, several authors have related the Chloroflexi overgrowth with bulking episodes, mainly in activated sludge reactors but also in full scale methanogenic reactors and lab-scale anammox reactors [30–34]. The study of Chloroflexi ecophysiology has direct implications in wastewater treatment plants performance, in particular to prevent and control bulking problems.

Here, we hypothesize that even though methanogenic, activated sludge and anammox reactors harbor different Chloroflexi species, their functions are akin. In order to test this hypothesis, we first performed 16S rRNA gene amplicon sequencing to determine the diversity of Chloroflexi in samples from anammox, activated sludge and methanogenic reactors. Then, we applied phylum-specific Fluorescence in situ hybridization (FISH) to determine the morphology of Chloroflexi in the different samples. Finally, we performed genome-centric metagenomics analysis to reveal their phylogenomic diversity and metabolic potential. The combination of different molecular approaches and the study of samples from different reactors allowed us to obtain information on the putative roles of Chloroflexi in different WWTS. Moreover, 17 new Chloroflexi species were identified and two of them are proposed as new Candidatus genus.

## Methods

### Sampling and DNA extraction

Samples from the following three reactors were analyzed (Table 1): 1) a full-scale upflow anaerobic sludge blanket (UASB) methanogenic reactor treating effluent from a malt industry (MO) ([35, 36], 2) a full-scale activated sludge reactor treating health-care waste treatment wastewater (Inffluent Chemical oxygen demand (COD_influent_) = 8,000 ppm, COD_effluent_ = 500 ppm, pH = 7.5, Hydraulic retention time (HRT) = 4.7 days), the wastes were sterilized by autoclave and the wastewater is generated in this process (RH), 3) a lab scale upflow anammox sludge blanket (UAnSB) reactor (S) fed with sewage after a partial nitrification system [37] (the inoculum used on this reactor was also included in the analysis). This inoculum was sludge from an anammox sequencing batch reactor (AM) fed with synthetic wastewater [38]. The full-scale reactors were located in Montevideo (Uruguay) and the lab-scale reactor was operated in the GENOCOV Research Group in Barcelona (Spain).

**Table 1:** Characteristics of the studied reactors

Reactor type	Configuration	Samples name	Sampling day^a^	Scale	Substrate	Condition
Aerobic	AS^b^/floccular biomass	RH4	0	Full-scale	Health-care waste treatment wastewater	Stable conditions for more than 6 years
		RH5	300			
		RH6	390			
Methanogenic	UASB^c^/granular biomass	MO1	0		Brewery-malt processing industry	Stable conditions for more than 12 years
		MO2	210			
		MO3	300			
Anammox	SBR^d^/granular biomass	AM	Inoculum	Lab scale	Synthetic wastewater	Stable conditions for more than 6 years
	UAnSB^e^/granular biomass	S1	81		Sewage after a partial nitrification system	81 days of operation (seeded with sludge from AM)
		S3				
		S5				

^a^For reactors RH and MO day 0 was considered the first day of sampling, for the anammox reactor S day 0 was considered the inoculation day

^b^*AS* Activated sludge

^c^*UASB* Upflow anaerobic sludge blanket

^d^*UAnSB* Upflow anammox sludge blanket

^e^*SBR* Sequencing batch reactor

All reactors were operated under mesophilic conditions. In order to apply the differential coverage binning approach to assemble genomes [39] several samples were taken from MO, RH and S (including AM used as inoculum of reactor S). Three samples taken at different time points were collected from the methanogenic (MO1, MO2 and MO3) and activated sludge reactors (RH4, RH5 and RH6). Another three samples were collected at different heights of the sludge bed from the UAnSB anammox reactor (S1, S3 and S5) and one from the sludge used as inoculum (AM).

Biomass samples were stored at -20 °C for sequencing workflows and fixed according to the protocol of Amann et al. [40] for FISH. Detailed procedures of DNA extraction and FISH are described in the Supplementary Material.

### Community profiling using 16S rRNA gene amplicon sequencing

The taxonomic composition of the communities was studied by amplicon sequencing of the 16S rRNA gene with primers for V4 region [41, 42]. The raw data analysis was performed using Quantitative Insights Into Microbial Ecology’ pipeline (QIIME2 2020.11 release) [43]. The sequences were classified using MiDAS 3.7 database [44]. Detailed procedures of primers, QIIME2 analyses, data visualization, and phylogenetic analysis of the amplicon sequences are described in the Supplementary Material.

### Metagenome sequencing

Metagenomes were shotgun sequenced by Illumina HiSeq 4000 platform (Macrogen, Seoul, Korea) using Library Kit TruSeq Nano DNA Kit (350 bp). The yield was approximately 5 Gb of raw short-read sequences per sample (2 × 100 bp).

### Genome assembly, binning and genome annotation

The global quality of the metagenomes reads was checked using FastQC (v0.11.8) [45]. The reads were trimmed to remove adapters and bases below a quality score of 25 using Trimmomatic (v0.39) [46]. The trimmed reads from each reactor (MO and RH, separately) were pooled and assembled using MEGAHIT (v1.1.4–2) [47] with minimum k-mer length 43, maximum k-mer 75, with steps of four. The three samples from the anammox reactor S1, S3 and S5 were pooled and analyzed together with sample AM which was used as inoculum. The contigs obtained shorter than 1000 bp were removed. Quality filtered reads for each metagenome were mapped to the co-assembly contigs (> 1000 bp) using Bowtie2 (v2.3.4.1) [48] with default parameters. Genome bins were recovered using MetaBAT2 (v2.12.1) [49]. The completeness levels and contamination of the bins were assessed using CheckM (v1.0.13) [50]. The bins with an estimated completeness > 50% and contamination < 10% were reclassified using Genome Taxonomy Database GTDB-Tk (version v1.3.0 and GTDB-Tk reference data version r95) [51] to determine if they belonged to the Chloroflexi phylum. Chloroflexi bins were reassembled using SPAdes 3.10.0 [52]. All resulting contigs of > 1,500 bp were clustered using ESOM tools (emergent self-organizing map) [53], on the basis of its tetranucleotide frequency to identify and extract contaminant contigs in each genome. GUNC [54] was used to detect chimerism and to assign the taxonomy of the contigs in MAGs. Contigs assigned to a different phylum of Chloroflexi were removed from the MAGs. The CSS (clade separation score) estimated with gunc was closer to 0 for all genomes. A CSS closer to a value of 0 indicates that a genome is free of contamination and all genes are assigned to the same taxonomy.

Statistics of the reassembled bin were obtained using QUAST v5.0.2 and CheckM lineage wf (v1.0.13). Genes encoding ribosomal RNAs (rRNA) were predicted using the Bacterial rRNA predictor (Barrnap, https://github.com/tseemann/barrnap). The Average Nucleotide Identities (ANI) and the Average Amino Acid Identity (AAI) between MAGs and isolated species were determined using FastANI [55] and EzAAI [56]. The Chloroflexi genomes retrieved from methanogenic, activated sludge and anammox reactors were named as MO, RH and AMX, respectively, with a number suffix.

Genome phylogenetic analyses were performed with the GTDB-Tk tool. Newick format tree file was uploaded onto iTOL, a web-based tool for annotating and editing trees [57].

### Metabolic analysis

Protein coding sequences (CDS) were determined with Prokka (1.14.5) [58, 59]. Predicted amino acid sequences were annotated with KOALA (KEGG Orthology And Links Annotation) for K number assignment of KEGG Genes [60] and Metaerg with KEGG, pfam, swiss-prot and tigrfams databases [61]. Pathways and genes completeness were represented as blocks with different colors in Figs. 4 and 5. Carbohydrate-active enzymes (CAZY) were determined using EnrichM v0.5.0 (https://github.com/geronimp/enrichM) with HMMs from dbCAN [62].

## Results and discussion

Applying a series of molecular biology tools, the diversity and metabolic potential of the phylum Chloroflexi was studied in three different wastewater treatment systems: a methanogenic reactor (anaerobic, C-removal), an activated sludge reactor (aerobic, C-removal) and an anammox reactor (anaerobic, N-removal).

### Diversity of Chloroflexi members in the different wastewater reactors according to 16S rRNA gene amplicon sequencing

**Fig. 1:**
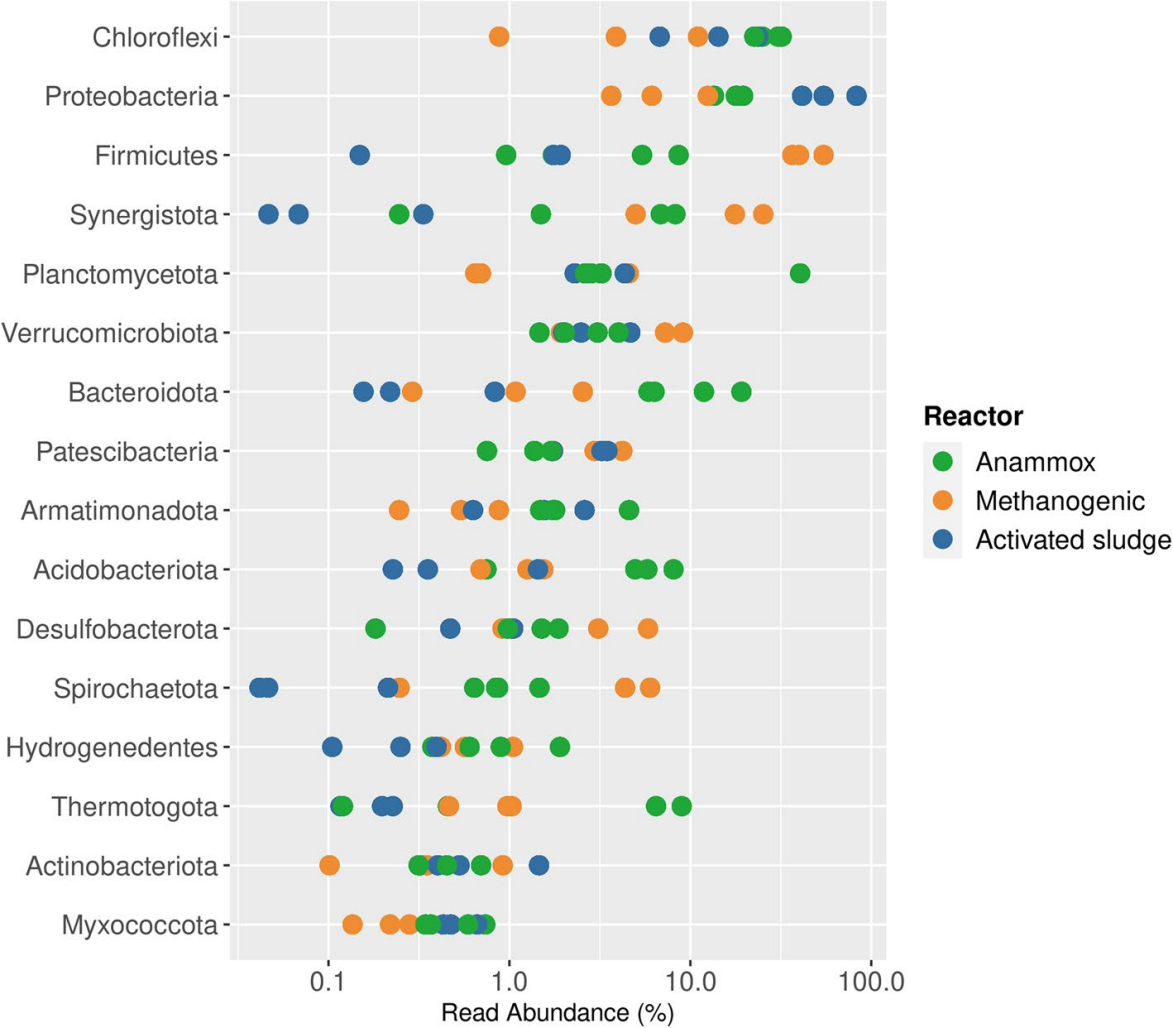
Boxplots showing the relative abundance of the 16 most abundant phyla in the total community according to the 16S rRNA gene amplicon sequence in each reactor type

The phylum Chloroflexi was detected in all reactor samples. Regarding the relative abundance higher values were detected in the anammox reactor samples compared to the methanogenic and aerobic reactors samples (26.9—33.5% for anammox, 4.9 -22% for activated sludge and 0.8—10.7% for methanogenic reactors) (Fig. 1).

**Fig. 2:**
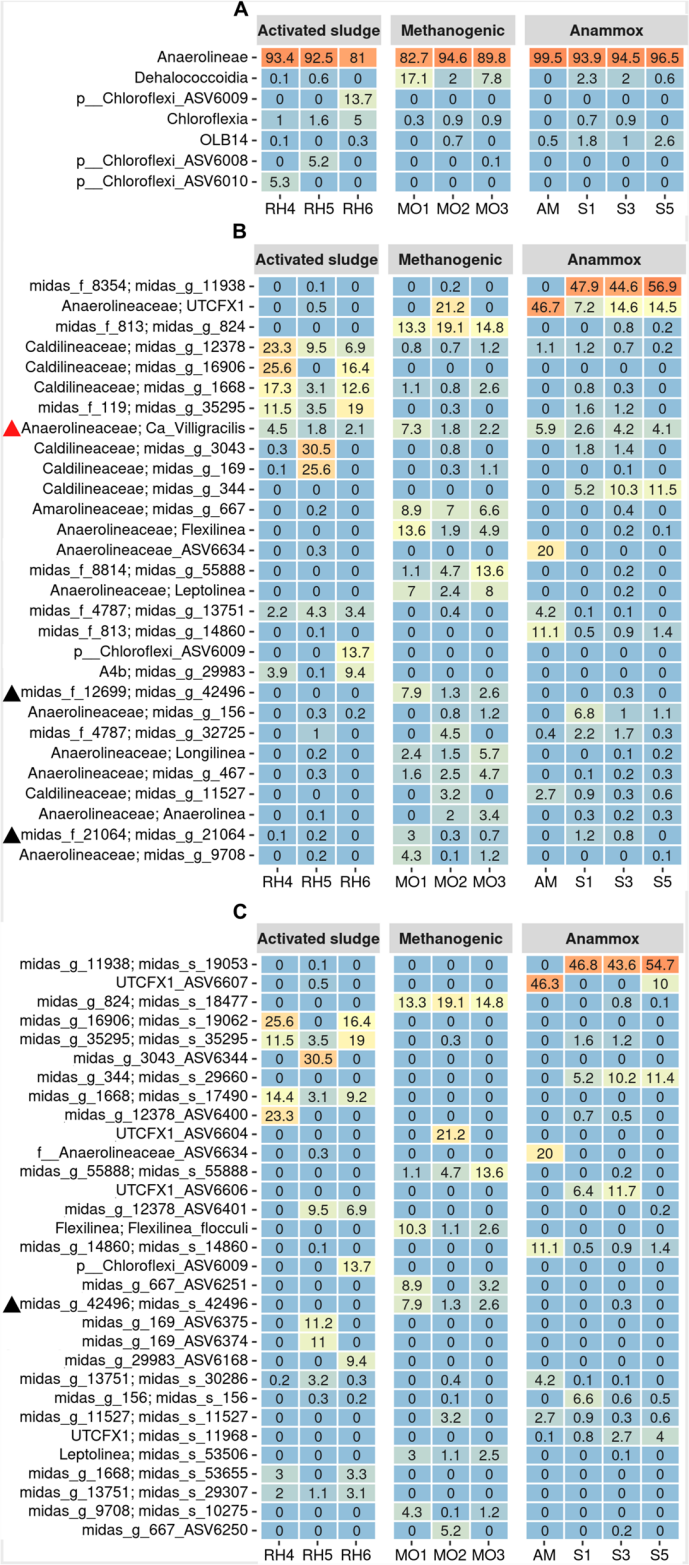
Heatmaps showing the relative abundance within the phylum Chloroflexi according to the 16S rRNA gene amplicon sequence: **A**) at class level, **B**) at genus level (30 most abundant genus) **C**) at species levels (30 most abundant species). Non-classified microorganisms are labeled with a number according to the MiDAS taxonomy. Black triangles in panels A and B mark genus and species belonging to Dehalococcoidia class, all the remaining microorganisms belonging to the class Anaerolineae. Red triangles in panel A mark ‘*Ca*. Villigracilis’

MiDAS database, specifically designed to analyze microbial communities from activated sludge systems and methanogenic reactors [63], was used to classify sequences at different taxonomic levels (non-described microorganisms are labeled with a number according to the MiDAS taxonomy). The results confirmed that Anaerolineae class widely dominated the Chloroflexi community in all reactors (Fig. 2a), which was in accordance with results previously reported in aerobic, anaerobic and anammox reactors [2, 4, 17, 26, 27, 29, 36, 64–68]. Most of the genera detected were specifically associated with one type of reactor.

One exception was ‘*Ca.* Villigracilis’, which was shared by all reactors with a similar relative abundance (Fig. 2b). This filamentous bacterium has been reported as widely distributed in activated sludge reactors, where it probably contributes to the matrix supporting floc formation [69]. Although ‘*Ca.* Villigracilis’ has been metabolically characterized by FISH-MAR and reported as the only facultative aerobic member described within Anaerolineae class so far, the genome is not yet available [69].

Several genera classified according to the MiDAS taxonomy, and therefore with no cultured representative, presented high abundance within the Anaerolineaceae and Caldilineaceae families in all reactors. Specifically, genera belonging to the family Caldilineaceae were the most abundant in the activated sludge reactor (Fig. 2b). Meanwhile, genera belonging to the family Anaerolineaceae were the most abundant in anammox and methanogenic reactors.

From all genera detected in our samples, only five harbor cultured representatives: *Flexilinea*, *Bellilinea*, *Longilinea*, *Leptolinea* and *Anaerolinea*. All of them have strains isolated from anaerobic reactors which were described as strictly anaerobic chemoorganotroph involved in carbohydrates fermentation [9, 10, 12]. In pure culture, their growth was enhanced in co-cultivation with a hydrogenotrophic methanogens. This could explain why sequences belonging to these genera were almost exclusively detected in the methanogenic reactor.

Moreover, all genera were represented by few dominant species which in most cases were not shared between reactors (Fig. 2c). In the samples studied, all Chloroflexi members presented filamentous morphology except for the anammox reactor samples (these results are described in the Supplementary material, section Morphology determined by FISH).

Several ASVs determined using the 16S rRNA gene sequences, could not be classified at class (e.g., ASV6009, ASV6008, ASV6010), genera (ASV6634) or species level (e.g., ASV6007, e.g., ASV6634, e.g., ASV6400, etc.). Even though MiDAS database represents a great advance in understanding the microbiomes of wastewater treatment systems, there are still undescribed microorganisms not included and more effort is needed to expand this database.

### General genomic features of Chloroflexi metagenomic assembled genomes

We used differential coverage binning [39] to obtain metagenomic assembled genomes (MAGs) from the three different reactors. A total of 75, 48 and 56 MAGs with > 50 completeness and < 10% contamination were successfully retrieved from MO, AMX and RH reactors samples, respectively. Seventeen MAGs belonging to the phylum Chloroflexi (9 AMX, 4 MO and 4 RH) were analyzed further. Recovered genomes completeness ranged from 85.59% and 96.79%, and contamination after the reassembly and polishing (manually curated) ranged between 0% and 3.36% (Table 2).

**Table 2:** Statistics summary for the 17 Chloroflexi MAGs recovered in this study

Reactor	Genome	Genome size (bp	CDS^a^	Completeness^b^/ Contamination	Contigs	N50 (bp)	16S/23S/5S^d^	tRNA^a^	%GC	Coverage	Taxonomic annotation^c^	Closest placement ANI %
Activated sludge	RH21	5,657,355	5097	85.59/0	655	12,275	1(922nt)/1(492nt)/1	4 5	56.34	8.2	c_Anaerolineae;o_SBR1031;f_A4b;g_OLB15	76.07
	RH38	5,163,972	4600	87.29/1.73	1167	5326	1(805nt)/1(342nt)/2	32	64.57	8.6	c_Anaerolineae;o_Caldilineales;f_Caldilineaceae	N/A
	RH43	9,980,378	8014	90.86/4.7	129	94,438	1(807nt)/1/1	44	55.88	3.03	c_Anaerolineae;o_Caldilineales;f_Caldilineaceae;g_CFX5	76.3
	RH52	4,922,116	3510	96.79/1.1	201	35,320	1(62nt)/1/1	57	73.22	20.4	c_Anaerolineae;o_UCB3;f_UCB3;g_UCB3;s_UCB3 sp003576755	99.2
Methanogenic	MO16	3,073,917	2871	86.36/3.36	387	11,990	1(1118nt)/0/0	35	58.98	10	c_Anaerolineae;o_Anaerolineales;f_Anaerolineaceae;g_Longilinea	79.35
	MO53	4,940,107	4005	87.27/0.91	227	44,487	1(337nt)/1(370nt)/1	45	58.31	13.5	c_Anaerolineae;o_B4-G1;f_SLSP01	N/A
	MO66	4,384,884	3803	92.73/3.36	478	14,973	0/0/0	44	64.91	14.7	c_Anaerolineae;o_UBA3071;f_CG2-30–64-16	N/A
	MO118	3,352,740	2734	90.18/2.73	86	51,993	1(847nt)/1(337nt)/1	38	41.05	13.2	c_Anaerolineae;o_Anaerolineales;f_Anaerolineaceae;g_Flexilinea	83.18
Anammox	AMX9	4,128,696	3450	98.18/0	54	178,058	1(800nt)/1(617nt)/1	48	62.69	32.7	c_Anaerolineae;o_SBR1031;f_A4b;g_OLB13;s_OLB13 sp001567485	99.66
	AMX14	3,390,571	3072	92.73/0.91	124	43,811	1(329nt)/0/0	41	56.87	25.1	c_Anaerolineae;o_Anaerolineales;f_EnvOPS12;g_OLB14;s_OLB14 sp900696595	99.72
	AMX15	3,646,596	3399	90.91/0.18	38	293,047	1(534nt)/2/1	42	54.08	160.3	c_Anaerolineae;o_Anaerolineales;f_EnvOPS12;g_UBA12294;s_UBA12294 sp003577395	95.27
	AMX39	2,675,910	2460	90.91/0.91	18	265,768	1(445nt)/0/0	48	52.31	19.8	c_Anaerolineae;o_Anaerolineales;f_EnvOPS12;g_UBA12294;s_UBA12294 sp002050275	99.2
	AMX47	2,814,255	2926	91.21/0	319	11,859	1(1498nt, 1518nt)/1/1	42	66.7	10.4	c_Dehalococcoidia;o_UBA2991;f_UBA2991;g_UCB2	82.41
	AMX55	2,990,515	2772	92.73/1.27	61	69,781	1(1509nt)/1(317nt)/1	40	60.81	15.1	c_Anaerolineae;o_Anaerolineales;f_EnvOPS12;g_UBA7227;s_UBA7227 sp002473085	98.72
	AMX56	4,499,265	3993	92.42/1.09	96	86,147	3(1218–1493)/2/2	58	63.48	54.1	c_Anaerolineae;o_Caldilineales;f_J102	76.67
	AMX57	4,700,319	4113	92.73/0	236	29,630	1(703nt)/1(461nt)/1	46	60.87	13.6	c_Anaerolineae;o_SBR1031;f_A4b;g_GCA-2702065	75.87
	AMX68	4,329,913	4110	92.73/0.91	70	171,406	1(195nt)/1(219nt)/1	46	53.34	15.8	c_Anaerolineae;o_Anaerolineales;f_EnvOPS12;g_OLB14	79.8

*N/A* Not assigned

^a^As predicted using Prokka (details in Methods section)

^b^Genome quality estimates from CheckM (details in Methods section)

^c^Taxonomic assignments from GTDB-Tk (details in Methods section)

^d^Sequences obtained from Barrnap (details in Methods section)

Three of these MAGs (AMX47, AMX55 and AMX56) were > 90% complete, had less than 5% contamination and importantly, included the full-length 16S, 23S, and 5S rRNA genes, and > 18 tRNA genes (Table 2), satisfying the criteria for high-quality (HQ) draft MAGs, according to the minimum information about a MAG (MIMAG) standard [70].

### Phylogenomic analysis of Chloroflexi MAGs

**Fig. 3:**
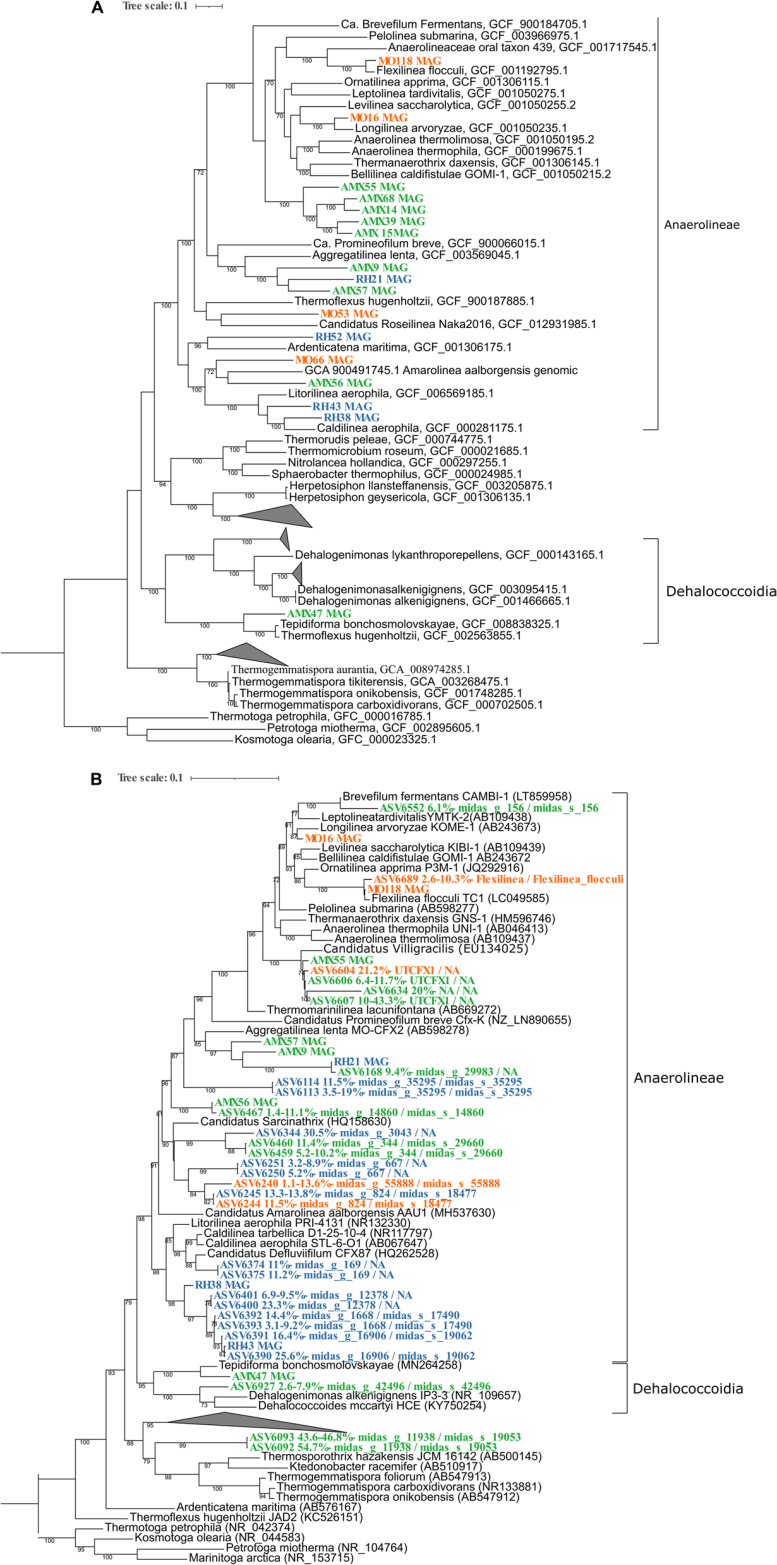
Phylogenomic and phylogenetic analysis of Chloroflexi based on the metagenomics results and the 16S rRNA gene sequence analysis: **A**) Phylogenomic tree constructed with the 17 genomes from this work and reference genomes retrieved from the NCBI, **B**) Phylogenetic tree constructed with the most abundant ASVs retrieved from the 16S rRNA gene amplicon sequences (with abundance higher than 5% in at least one sample) and the sequences of the 16S rRNA gene from 10 MAGs from this work, and reference sequences of the phylum Chloroflexi retrieved from NCBI. The tree was reconstructed using the ML method and the GTR model. ML bootstrap values greater than or equal to 70% are shown at each node. Bar indicates 0.01 substitutions per site. The ASV and MAGs are colored by reactor: blue for RH, orange for MO and green for AMX. Sequences from the phylum Thermotogota were used as outgroup for rooting trees

To determine the phylogenetic position of the MAGs, a phylogenomic tree based on concatenated alignments of 120 single copy marker genes was constructed using 105 reference Chloroflexi genomes retrieved from NCBI (February 2021) and the 17 Chloroflexi MAGs obtained in our work. Of the 17 MAGs, six had sufficiently high degree of similarity (> 95% ANI to a representative genome [71]) to be classified at species level. The remaining MAGs presented ANI values below 95% with any reported genome and could not be classified at species level. According to the phylogenomic analysis, 7 were classified to genus level and 4 to family level (Table 2). The results showed that most of the MAGs (16 MAGs) were positioned into the Anaerolineae class while only one genome was positioned within the Dehalococcoidia class (Fig. 3a).

At the order level, MAGs were distributed within the orders Anaerolineales, SBR1031, B4-G1, Caldilineales, UBA3071, UCB3 and UBA2991 (Table 2).

In addition, a phylogenetic tree was constructed based on the 16S rRNA gene sequences retrieved from 10 MAGs and the ASV sequences retrieved from the amplicon sequencing analysis (Fig. 3b, Fig. S2). Because of the few genomes available in the databases, it was not possible to construct both phylogenetic trees (based on 16S rRNA gene sequences and based on genomes sequences) with the exact same members of Chloroflexi. For example, ‘*Ca*. Villigracilis’ and ‘*Ca*. Sarcinatrix’, do not have representative genomes in databases so far, and were therefore not added to the genome-based phylogenetic tree. Moreover, although frequently used for phylogenetic identification, 16S rRNA gene sequences are notoriously difficult to assemble from metagenomes [72]. Thus, 16S rRNA genes were present only in 10 of the 17 MAGs (Table 2). Taking all this into consideration, the phylogenetic placement of 10 MAGs based on the 16S rRNA gene (Fig. 3b) was consistent with the phylogenomic tree based on 120 marker genes genes (Fig. 3a).

Interestingly, five MAGs from the anammox reactor (AMX55, AMX15, AMX39, AMX14 and AMX68), formed a monophyletic clade separated from other genomes in the phylogenomic tree (Fig. 3a). The 16S rRNA gene from AMX55 clustered with the sequence of *‘Ca.* Villigracilis’ in the phylogenetic tree, with a 95.68% of identity according to the MiDAS taxonomy (Fig. 3b). Therefore, the retrieved MAG represents the first recovered genome within the ‘*Ca.* Villigracilis’ genus [73].

Within the ‘*Ca*. Villigracilis’ cluster, an ASV was classified within the UTCFX1 genus according to MiDAS. UTCFX1 represents a MAG retrieved from a nitritation-anammox sequencing batch reactor. The authors detected the presence nitrate reduction genes in this genome which were actively transcribed according to the metatranscriptomics analysis suggesting an important interaction with anammox microorganisms [26].

### New genera and species candidates

In total, three MAGs (AMX47, AMX55 and AMX56) satisfied the criteria for high-quality recovered genomes [70] and met the minimal suggested required standards to be proposed as *Candidates* of new genera or species [74] (Table 2, Table S1). The full-length 16S rRNA gene sequence from AMX47, AMX55 and AMX56 showed identity values between 90.14% and 96.99% with their closest relatives according to Silva and MiDAS database comparisons (Table S2). The three MAGs presented ANI values < 79.2% with genomes from described species, and the AAI values for AMX47, AMX55 and AMX56 were 63.1, 57.6 and 56.6%, respectively (Table S1). Given the lack of close relatives, AMX47 and AMX56 genomes represent two different novel genera in the phylum, meanwhile, AMX55 represents the first representative genome of the genus ‘*Ca*. Villigracilis’ (based on the taxonomic sequence identity threshold recommendations of [75].

### General analysis of metabolic pathways and genes

Different Chloroflexi genera and species predominate in the different wastewater treatment systems studied but, is their function redundant? For the 17 Chloroflexi MAGs, genes were annotated using a variety of protein databases to infer their metabolic potential. Because most of these MAGs were estimated to be between 85 and 93% complete, genes for additional pathways, might not be identified in this study. To overcome this problem, we also annotated genomes from described species closely related to our assembled genomes according to the phylogenomic tree (Fig. 3a). This allowed us to infer if a metabolic pathway of interest was not present in the genome or if its absence might be due to an incomplete genome recovery.

The metabolic pathway analysis was focused on answering the following questions:

- 1-Are they potentially capable of performing aerobic and/or anaerobic respiration?
- 2-Do they have the potential capability to hydrolyze different compounds?
- 3-Which carbon compound degradation pathways do they have?
- 4-Do they have N-removal potential?
- 5-Do they have genes related to biomass adhesion properties?

### Aerobic and anaerobic respiration pathways

Reactors were operated in anaerobic (anammox and methanogenic reactors) or aerobic (activated sludge reactor) conditions and therefore, genes related to aerobic and anaerobic respiration, or fermentative metabolisms were searched against the assembled MAG sequences.

**Fig. 4:**
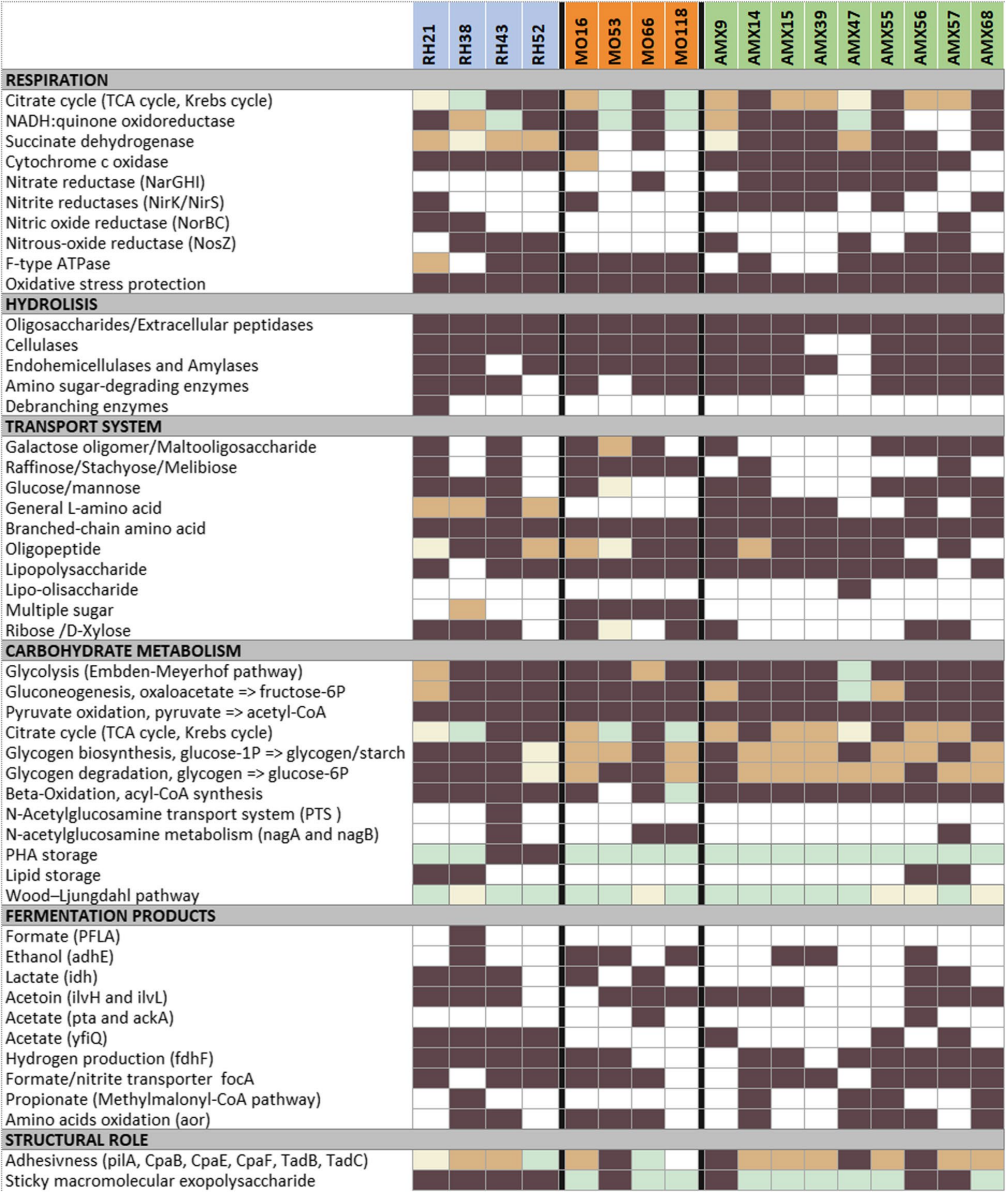
Heatmap showing the completeness and the incompleteness of each metabolic pathway for the 17 MAG. Colors indicate pathways level completeness: dark brown (complete), light brown (1 block missing), beige (2 block missing), light green (more than 2 blocks missing) and white (not present)

Regarding respiration pathways, the genomic analysis showed that most MAGs (10 out of 17) had an incomplete oxidative phosphorylation pathway. Five MAGs (RH43, RH52, AMX55, AMX14 and AMX56) harbor the genes to use O_2_ and different nitrogenous compounds (N_2_O, NO_3_^−^ and/or NO_2_^−^) as final electron acceptors, and two of them presented only the genes to use NO_3_^−^ or NO_2_^−^ (MO66 and AMX68, respectively) (Fig. 4, Fig. S3, Table S1).

The complete phosphorylation pathway and the presence of nitrate and nitrite reductases in AMX55 was in accordance with the in situ characterization of ‘*Ca*. Villigracilis’, as this genus has the ability to take up substrates under anoxic conditions in presence and absence of nitrate/nitrite [69]. As we mentioned, the metatranscriptomics analysis showed that nitrate reduction genes from UTCFX1 (closely related to AMX55 in the phylogenetic tree, Fig. 3b) were expressed in an anammox reactor [26].

The tolerance to aerobic conditions is determined by the presence of oxidative stress protection genes. The presence of these genes was expected in genomes retrieved from the activated sludge reactor as these microorganisms are continuously exposed to oxygen (RH21 and RH38). Nevertheless, these genes were found in all MAGs (Fig. 4, Table S1). This is in concordance with the results obtained for ‘*Ca.* Brevefilum fermentans’ retrieved from an anaerobic digester [17].

### Metabolic pathways involved in polymers hydrolysis, carbon respiration and fermentation

To test the hypothesis that Chloroflexi members are capable of recycling soluble microbial products acting as hydrolytic bacteria, we performed the annotation of genes for glycosyl hydrolases such as cellulases, endohemicellulases, amylases, amino sugar-degrading enzymes, oligosaccharide-degrading enzymes, and also extracellular peptidases. The presence of all these genes indicated that most MAGs have the potential of hydrolyzing cellulose, starch, protein and/or peptides (Fig. 4, Supplementary Data 1). This is in accordance with previous in situ studies, which revealed high level of surface associated hydrolytic enzymes and their involvement in the breakdown of complex organic compounds [14, 76]. This result indicates that the Chloroflexi phylum may play an active role in hydrolyzing complex organic matter in activated sludge, as well as in methanogenic and anammox reactors.

Regarding central carbon metabolism, all MAGs contained multiple transporters for different organic compounds (including sugars, amino acids, proteins and fatty acids) indicating that each species has alternative routes for incorporating and recycling dissolved organic matter (scavenge macronutrients) (Fig. 4, Supplementary Data 1). The Embden-Meyerhof-Parnas (EMP) pathway for glycolysis was complete in all MAGs except in RH21, MO66 and AMX47. RH21 and MO66 are phylogenetically related to *Aggregatilinea lenta* and *Litorilinea aerophila*, respectively, which have the complete glycolysis pathway. Thus, incomplete glycolysis pathways in RH21 and MO66 genomes may be due to incomplete genome recoveries. On the other hand, AMX47 was closely related to *Dehalococcoides mccartyi*, which does not have the complete glycolysis pathway suggesting that AMX47 does not perform glycolysis. The potential of AMX55 to consume glucose was consistent with the results showed for ‘*Ca*. Villigracilis’ [69]. Genes for pyruvate oxidation to acetyl-CoA were annotated in all MAGs (Fig. 4, Supplementary Data 1). Beta-oxidation was annotated in most of the MAGs and might represent an important metabolic route to obtain carbon and reducing equivalents for all Chloroflexi species. Another interesting finding was that genes for N-acetylglucosamine transportation and metabolism (PTS, *nag*AB) were annotated in RH43 (Fig. 4, Supplementary Data 1). These results are in accordance with other reports where Chloroflexi members appear to retrieve N-acetylglucosamine from lysed cells revealed by micro-autoradiography or FISH studies [3, 14, 23, 77–82]. In addition to the N-acetylglucosamine metabolism, we suggest that scavenging occurs through hydrolysis of complex organic compounds outside the cell, which are then transported to the cytoplasm, and are then metabolized via glycolysis or beta-oxidation (degradation of fatty acids and branched-chain amino acids) pathways. These results support the previous hypothesis that Chloroflexi has an important beneficial role in degradation of lysed bacterial cell debris and EPS.

Fermentation pathways including genes for acetate, ethanol, lactate, acetoin, formate and/or propionate production were present in all MAGs (Fig. 4, Supplementary Data 1). These results were in accordance with previous information of the Anaerolineae class (isolates and MAGS) retrieved from wastewater treatment system which were involved in sugar, amino acid or protein fermentation with different end products [8–10, 12].

Metabolic pathways for amino acids degradation and propionate formation (methylmalonyl-CoA pathway and aldehyde:ferredoxin oxidoreductase) was found in five MAGs (RH38, AMX14, AMX47, AMX55 and AMX68). These genes were also annotated in other Chloroflexi members [17, 83–85].

Most of the MAGs (13 MAGs) contained a formate dehydrogenase H (*fdh*F) to convert formic acid decomposition into CO_2_. Only AMX55 had the ability to convert formic acid into H_2_ and CO_2_ by the presence of both *fdh*F and hydrogenases, under anaerobic conditions in the absence of exogenous electron acceptors as was observed in previous studies [82, 86]. In addition, the formate transporter (*foc*A) was annotated in most of these MAGs. Synergistic interaction of Anaerolineae members with the methanogenic archaea, was previously reported in anammox [87] and methanogenic reactors [2, 17, 88]. This could be a common scenario in these systems where the excess of electrons from organic carbon oxidation by Anaerolineae members could be transferred to methanogenic archaea.

### Potential for polymers and lipids storage

The potential to store glycogen, polyhydroxyalkanoates and lipids was more common among MAGs retrieved from activated sludge than from methanogenic or anammox reactors. RH21, RH38, RH43, MO66 and AMX9 had complete glycogen biosynthesis and degradation pathways, suggesting that this polysaccharide may serve as a possible storage compound under unbalanced growth conditions (e.g., when C and/or N is temporally limited) (Fig. 4, Supplementary Data 1) [89, 90]. This result is in accordance with the information reported for ‘*Ca.* Amarolinea aalborgensis’ and ‘*Ca.* Promineofilum breve’ both retrieved from activated sludge [7, 16].

In addition to glycogen as storage compound, RH43 and RH52, encodes a potential biosynthesis pathway for polyhydroxyalkanoates (PHA) (Fig. 4, Supplementary Data 1), which are usually formed as carbon and energy storage compounds [91] under conditions of carbon excess and nitrogen or phosphate limitation [92]. Thus, the potential for PHA storage could favor these organisms with intermittent carbon availability present in these systems. Also, the annotation of a putative acyl-CoA:DAG acyltransferases (*atf*A), which catalyzes the final step in the synthesis of triacylglycerols, indicates the potential for lipid storage [93] in RH21, RH38, AMX56 and AMX57. This capability was also reported for ‘*Ca.* Promineofilum breve’ [16].

### Presence of Nitrogen metabolism pathways

**Fig. 5:**
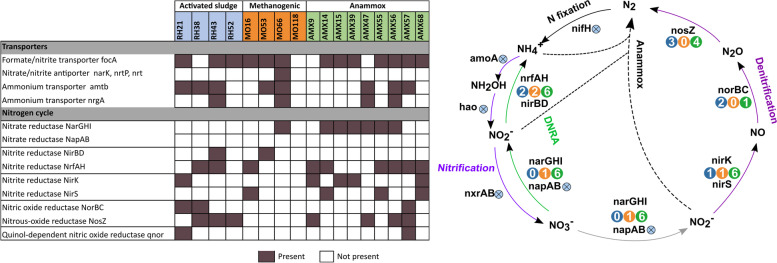
Heatmap showing the nitrogen cycle annotated genes in the 17 MAGs from activated sludge, methanogenic and anammox reactors, Nitrogen cycle, next to each gene, the number of genomes that contain that gene is shown. Blue: activated sludge. Orange: methanogenic. Green: anammox. Dark brown blocks indicate genes that are present and white blocks indicate genes that are not present

Different genes related to the nitrogen cycle such as the dissimilatory nitrate reduction to ammonia (DNRA) and partial denitrification were mostly annotated in Chloroflexi MAGs from anammox reactors. DNRA is part of the nitrogen cycle, and it has been annotated in several Chloroflexi genomes [7, 16, 26]. In this work, three Chloroflexi species from anammox reactors (AMX14, AMX55 and AMX56) have the potential capability to perform DNRA due to the presence of both dissimilatory nitrate (*nar*GHI) and nitrite reductases (*nrf*AH) (Fig. 5).

AMX14 and AMX55 genomes were positioned in the ‘*Ca*. Villigracilis’ and UTCFX1 cluster. As mentioned above, UTCFFX1 had nitrate reductase genes activity [26]. Thus, probably both species might perform DNRA. The six remaining MAGs have the potential to reduce nitrate to nitrite (*nar*GHI), or nitrite to ammonium (*nrf*AH). Our results show that all MAGs obtained from the anammox reactor could have a specific role in the nitrogen cycle, supplying nitrite or ammonia to anammox bacteria enhancing the overall nitrogen removal performance.

Two MAGs retrieved from the activated sludge reactor harbor genes to perform the reduction of nitrite to ammonia. These results are in accordance to those reported for ‘*Ca.* Amarolinea’ (Chloroflexi MAG retrieved from activated sludge) [7]. Despite its potential, the in situ characterization did not confirm the use of nitrate or nitrite [18]. Also, putative nitrite reductase (*nrf*AH) has been found in *Caldilinea aerophila* and *Anaerolinea thermophile* genomes, but their ability to utilize nitrite as an electron acceptor to support anaerobic growth is yet to be assessed [8].

A nitrite reductase and nitric oxide reductase were annotated in RH21, suggesting the potential to denitrify (Fig. 5). This ability has only been proved for one Chloroflexi species, *Ardenticatenia maritima* [94]. In the anammox reactor, the presence of *nir*K/*nir*S and *nos*Z genes was common among the assembled MAGs. It was consistent with several studies which report that denitrification is more frequent in Chloroflexi species from anammox reactors than in the ones retrieved from aerobic and anaerobic reactors [26, 27, 68]. A recent metagenomic study revealed that most of the heterotrophic organisms in anammox granules encode the ability to respire nitrate via partial denitrification, possibly completing a nitrite loop with anammox and nitrite oxidizing bacteria (NOB) by reducing nitrate back to nitrite [68]. This activity could contribute to the removal of excess nitrate produced from the system during anammox growth or nitrite oxidation by NOB.

### Filamentous morphology, adhesion capability and exopolysaccharides production

It has been extensively proposed that Chloroflexi plays an important role in granule and floc formation. In order to deepen our knowledge on this subject, three important capabilities were specifically searched in the MAGs: the filamentous morphology (studied by FISH), the adhesiveness and the production of exopolysaccharides. A complete set of genes for the pilus assembly (pilA, CpaB, CpaE, CpaF, TadB, TadC) which favor adhesiveness, was annotated in MO53, AMX9, AMX47 and AMX56 (Fig. 4, Supplementary Data 1). The other MAGs mainly missed only the pilus assembly protein CpaB. As has been already noted, pili are often involved in facilitating adhesion and colonization in a wide variety of scenarios. In a previous study of Anaerolineae members, the adhesiveness by the expression of the tight adherence protein (Tad) on the active type VI pili indicated its function for cellular attachment, which was further testified to be more likely related to cell aggregation other than cellulose surface adhesion [29].

Regarding the production of exopolysaccharides, it has been suggested that some Chloroflexi-affiliated bacteria encode the function of biosynthesizing sticky macromolecular exopolysaccharides such as UDP-GlcNAcA, GDP-Man, and GDP-Rha from partial nucleotide sugars biosynthesized by anammox bacteria (UDP-ManNAc and CDP-Glc) [27]. We found the same set of genes for sticky macromolecular exopolysaccharide synthesis in all MAGs from RH, 3 MAGs from AMX and 1 MAG from MO, including the enzyme lactate dehydrogenase (prerequisite for exopolysaccharide production) (Fig. 4, Supplementary Data 1). The rest of the MAGs lack some of the necessary genes. Therefore, the results obtained showed that some Chloroflexi have the capability to promote cell aggregation and consequently the formation of cores or carriers, which help to form the initial framework of small sludge particles. Zhao et al. [87] reported that Anaerolineae members affected the nitrogen removal performance through affecting the aggregation because of the positive correlation relationship of this group with the abundance of EPS formation genes. The results obtained showed that key genes involved in B-vitamin biosynthesis were missing in all Chloroflexi MAGs. For example, genes for thiamin (vitamin B1) biosynthesis (thiamine-phosphate synthase and thiamine-monophosphate kinase), biotin (vitamin B7) biosynthesis (adenosylmethionine-8-amino-7-oxononanoate aminotransferase and biotin synthase) and adenosylcobalamin (vitamin B12) biosynthesis (cobalamin synthase and adenosylcobinamide-phosphate synthase) were absent in all Chloroflexi MAGs (Supplementary Data 1) as previously reported by Lawson et al. 2017 [95]. These authors postulated that in anammox reactors *Brocadia* sp. could supply B-vitamins to Chloroflexi members. This suggests that other microorganisms may support B-vitamin requirements for Chloroflexi community. This hypothesis could be extended to activated sludge and methanogenic reactors.

### The role of Chloroflexi species in each reactor

The role of the different MAGs was postulated according to the genome annotation and the reactor´s characteristics (Table 3).

**Table 3:** Role proposed for the Chloroflexi microorganisms in the different reactors according to the metabolic function detected in the MAGs

Reactor	Process	Wastewater composition	Metabolic role	MAGs harvoring this function	Structure role in biomass
MO	Anaerobic treatment for C removal	Polysaccharides, proteins, carbohydrates	Hydrolysis of complex polymers from the wastewater by fermentation, EPS degradation	All MAGs	Granules structure
RH	Aerobic treatment for C-removal	Polysaccharides, proteins, carbohydrates	Hydrolysis of complex polymers from the wastewater by fermentation or aerobic respiration, EPS degradation	All MAGs	Flocs structure
S and AM	N-removal by anammox	Ammonium and nitrite	EPS and cell debris degradation	All MAGs	Anammox granules structure
			Reduction of nitrate	AMX14, AMX15, AMX39, AMX47, AMX55, AMX56	
			Reduction of nitrite	AMX9, AMX14, AMX55, AMX56, AMX57, AMX68	

Although the reactors were fed with very different wastewaters and operated under different conditions (anaerobic, aerobic and anoxic) a common role can be postulated in the degradation of polymers (polysaccharides or proteins) either from the wastewater or from biomass decay or exopolymers secreted by cells. Another important role can be also postulated in the maintenance of cell aggregates as granules or floccules. Yet, Chloroflexi species can grow by using a fermentative way of life (anaerobic), aerobic respiration or nitrate-nitrite reduction, depending on the reactor’s operation.

In conclusion, although the information retrieved from the metagenomics analysis showed shared functions in the different reactors, adaptation to each operation condition was observed, indicating high versatility within this group of microorganisms.

### General Chloroflexi features and metabolism

**Fig. 6:**
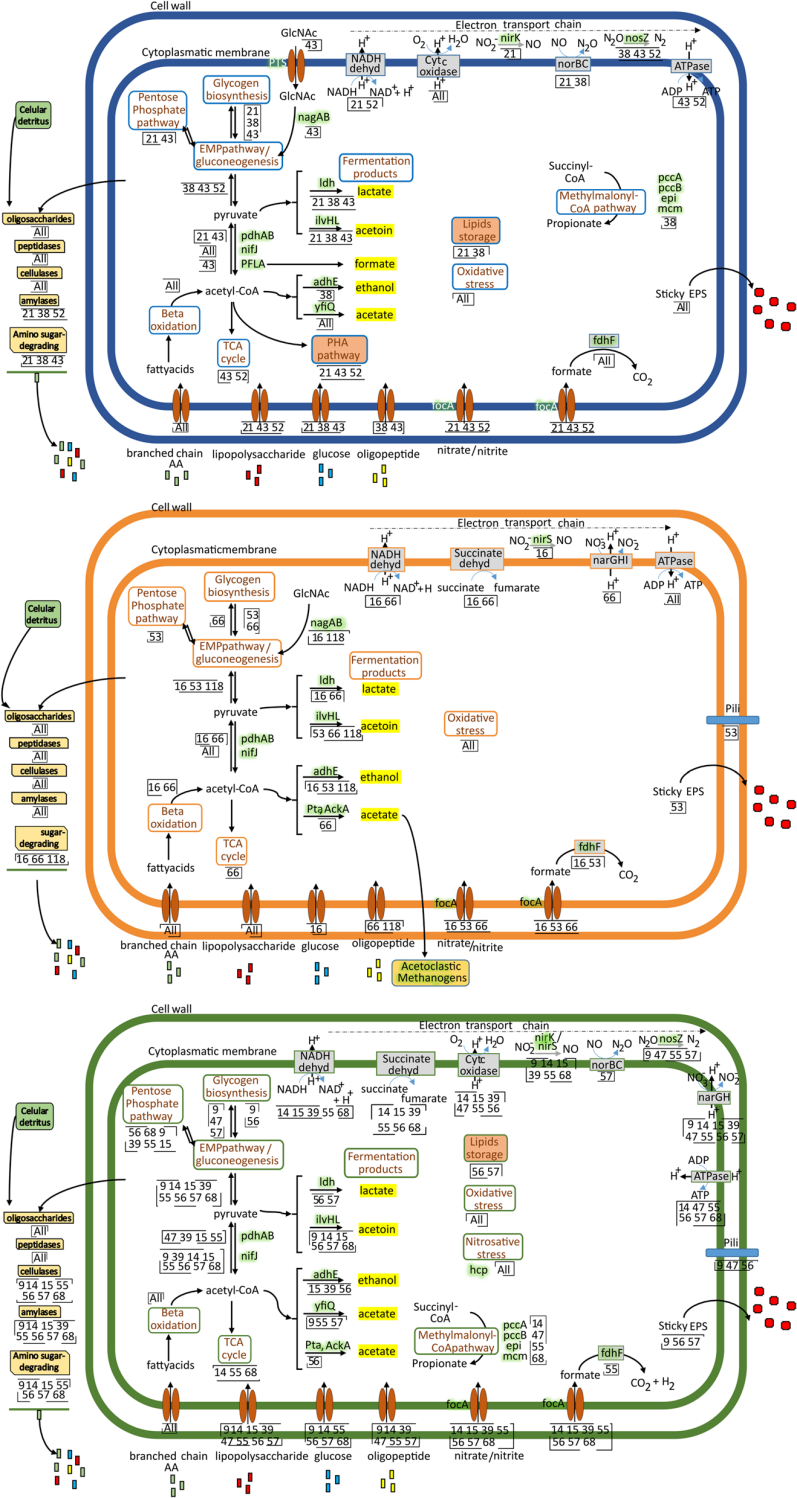
Metabolic model of the Chloroflexi MAGs by reactor: Blue: MAGs retrieved from the aerobic reactor, Orange: MAGs retrieved from the methanogenic reactor, Green: MAGs retrieved from the anammox reactor and its inoculum. The identifying number of each MAG appears in the boxes when the gene or metabolic pathway is present

A summary of the metabolic and structural functions from the MAGs retrieved from the three ecosystems is shown in Fig. 6 and Table 3.

The genomic annotation of the 17 MAGs revealed the potential for facultative and strict metabolism. All MAGs had the potential of a versatile metabolism related to the hydrolysis and fermentation of complex and simple organic compounds, suggesting that these hydrolytic and fermentative bacteria occupy a niche in recycling microbial dissolved organic matter. In addition to the filamentous morphology, some species could have a crucial structural role providing the structural matrix around which floc and granules material aggregates due to their adhesiveness and EPS production. Also, these characteristics could represent a selective advantage for the retention of these bacteria in the reactors.

Although the MAGs were retrieved from very different ecosystems, there was functional redundancy in the same reactor and analogous functions between reactors related to carbohydrates metabolism, and a more specific role related to the nitrogen removal in anammox reactors (Fig. 6). The functional redundancy of Chloroflexi members enables functional resistance to reactor operation disturbances due to the presence of multiple species that can perform the same metabolic function.

While MAGs analysis expanded our knowledge on the diversity and potential function of the phylum Chloroflexi, further experiments are necessary to confirm the expression of the identified metabolic functions under certain operational conditions. On the other hand, studying gene expression in bulking episodes would deepen the knowledge about their implication in these events. This knowledge would allow us to understand further which Chloroflexi groups are functionally important in these systems and how their disappearance or overgrowth could affect the processes. And as a final goal manage them and for example reduce and control bulking events. The difficulty of isolating organisms of the phylum Chloroflexi are probably related to their slow growth. New tools have been developed that facilitate the isolation of slow-growing microorganisms such as Droplet-based high-throughput cultivation [96]. Therefore, it is expected in the coming years to have a greater quantity of Chloroflexi members isolated in pure culture, in order to reveal and confirm their role in wastewater treatment systems.

### Taxonomic proposal for the new Candidates

#### ‘*Ca.* Villigracilis nielsenii’ sp. nov.

As a name for the new candidate species (represented by the AMX55 genome) within the genus ‘*Ca*. Villigracilis’, we suggest ‘*Ca.* Villigracilis nielsenii’. Niel.se’ni.i. N.L. gen. masc. n. nielsenii, of Nielsen, named in honor of Per Halkjær Nielsen, the Danish environmental microbiologist who made important contributions to research and practices in the field of Microbial Ecology and Water Engineering.

## Conclusion

This study provides a first insight into the diversity and metabolic potential of 17 Chloroflexi species in three different wastewater treatment bioreactors, unveiling the ecological role of many species with no representatives in pure culture. The recovered genomes appear in several taxonomic orders, attesting to the broad diversity of Chloroflexi members in WWTS.

Our results suggest that Chloroflexi participate in organic matter degradation, nitrogen removal and biofilm aggregation, playing different roles according to the environmental conditions. Additionally, we proposed two novel genera within the classes Dehalococcoidia and Anaerolineae, and a new species of the genus ‘*Ca*. Villigracilis’ representing the first representative genome within this genus.

## Supplementary Information

**Additional file 1. **

**Additional file 2. **

## Data Availability

The raw amplicon and metagenome sequences and the 17 MAGs were deposited under the NCBI BioProjects: PRJNA728853 and PRJNA780299. Scripts are available in GitHub (https://github.com/PatoUru/Bovio-Winkler_etal_2022/blob/main/Bovio-Winkler_etal_2022).

## Abbreviations

WWTS: Wastewater treatment systems
SMP: Soluble microbial products
EPS: Extracellular polymeric substances
FISH: Fluorescence in situ hybridization
UASB: Upflow anaerobic sludge blanket
UAnSB: Upflow anammox sludge blanket
MAGs: Metagenomic assembled genomes
ANI: Average Nucleotide Identities
AAI: Average Amino Acid Identity
MIMAG: Minimum information about a MAG
ASV: Amplicon sequence variant

## References

[1] Petriglieri F, Nierychlo M, Nielsen PH, McIlroy SJ (2018). In situ visualisation of the abundant Chloroflexi populations in full-scale anaerobic digesters and the fate of immigrating species. PLoS ONE.

[2] Bovio-Winkler P, Cabezas A, Etchebehere C, Chandran K H, Lee PK, Halkjaer Nielsen P (2021). Database Mining to Unravel the Ecology of the Phylum Chloroflexi in Methanogenic Full Scale Bioreactors. Front Microbiol.

[3] Kindaichi T, Yuri S, Ozaki N, Ohashi A (2012). Ecophysiological role and function of uncultured Chloroflexi in an anammox reactor. Water Sci Technol.

[4] Cao S, Du R, Li B, Ren N, Peng Y (2016). High-throughput profiling of microbial community structures in an ANAMMOX-UASB reactor treating high-strength wastewater. Appl Microbiol Biotechnol.

[5] Wang S, Guo J, Lian J, Ngo HH, Guo W, Liu Y (2016). Rapid start-up of the anammox process by denitrifying granular sludge and the mechanism of the anammox electron transport chain. Biochem Eng J.

[6] Speirs LBM, Dyson ZA, Tucci J, Seviour RJ (2017). Eikelboom filamentous morphotypes 0675 and 0041 embrace members of the Chloroflexi: resolving their phylogeny, and design of fluorescence in situ hybridisation probes for their identification. FEMS Microbiol Ecol.

[7] Andersen MH, McIlroy SJ, Nierychlo M, Nielsen PH, Albertsen M (2019). Genomic insights into Candidatus Amarolinea aalborgensis gen. nov., sp. nov., associated with settleability problems in wastewater treatment plants. Syst Appl Microbiol.

[8] Sekiguchi Y, Yamada T, Hanada S, Ohashi A, Harada H, Kamagata Y (2003). Anaerolinea thermophila gen. nov., sp. nov. and Caldilinea aerophila gen. nov., sp. nov., novel filamentous thermophiles that represent a previously uncultured lineage of the domain bacteria at the subphylum level. Int J Syst Evol Microbiol.

[9] Yamada T, Imachi H, Ohashi A, Harada H, Hanada S, Kamagata Y (2007). Bellilinea caldifistulae gen. nov., sp. nov and Longilinea arvoryzae gen. nov., sp. nov., strictly anaerobic, filamentous bacteria of the phylum Chloroflexi isolated from methanogenic propionate-degrading consortia. Int J Syst Evol Microbiol.

[10] Yamada T, Sekiguchi Y, Hanada S, Imachi H, Ohashi A, Harada H (2006). Anaerolinea thermolimosa sp. nov., Levilinea saccharolytica gen. nov., sp. nov. and Leptolinea tardivitalis gen. nov., sp. nov., novel filamentous anaerobes, and description of the new classes Anaerolineae classis nov. and Caldilineae classis nov. in the. Int J Syst Evol Microbiol.

[11] Yoon DN, Park SJ, Kim SJ, Jeon CO, Chae JC, Rhee SK (2010). Isolation, characterization, and abundance of filamentous members of Caldilineae in activated sludge. J Microbiol.

[12] Sun L, Toyonaga M, Ohashi A, Matsuura N, Tourlousse DM, Meng XY (2016). Isolation and characterization of Flexilinea flocculi gen. nov., sp. nov., a filamentous, anaerobic bacterium belonging to the class Anaerolineae in the phylum Chloroflexi. Int J Syst Evol Microbiol.

[13] Yamada T, Sekiguchi Y (2009). Cultivation of uncultured chloroflexi subphyla: significance and ecophysiology of formerly uncultured chloroflexi “subphylum i” with natural and biotechnological relevance. Microbes and environments / JSME.

[14] Kragelund C, Levantesi C, Borger A, Thelen K, Eikelboom D, Tandoi V (2007). Identity, abundance and ecophysiology of filamentous Chloroflexi species present in activated sludge treatment plants. FEMS Microbiol Ecol.

[15] Nielsen PH, Kragelund C, Seviour RJ, Nielsen JL (2009). Identity and ecophysiology of filamentous bacteria in activated sludge. FEMS Microbiol Rev.

[16] McIlroy SJ, Karst SM, Nierychlo M, Dueholm MS, Albertsen M, Kirkegaard RH (2016). Genomic and in situ investigations of the novel uncultured Chloroflexi associated with 0092 morphotype filamentous bulking in activated sludge. ISME J.

[17] McIlroy SJ, Kirkegaard RH, Dueholm MS, Fernando E, Karst SM, Albertsen M, et al. Culture-independent analyses reveal novel anaerolineaceae as abundant primary fermenters in anaerobic digesters treating waste activated sludge. Frontiers in microbiology. 2017;8:1134. 10.3389/fmicb.2017.01134 PMC5481317 28690595

[18] Nierychlo M, Miłobȩdzka A, Petriglieri F, McIlroy B, Nielsen PH, McIlroy SJ (2019). The morphology and metabolic potential of the Chloroflexi in full-scale activated sludge wastewater treatment plants. FEMS Microbiol Ecol.

[19] Yamada T, Sekiguchi Y, Imachi H, Kamagata Y, Ohashi A, Harada H (2005). Diversity, localization, and physiological properties of filamentous microbes belonging to Chloroflexi subphylum I in mesophilic and thermophilic methanogenic sludge granules. Appl Environ Microbiol.

[20] Yamada T, Yamauchi T, Shiraishi K, Hugenholtz P, Ohashi A, Harada H (2007). Characterization of filamentous bacteria, belonging to candidate phylum KSB3, that are associated with bulking in methanogenic granular sludges. ISME J.

[21] Strous M, Heijnen JJ, Kuenen JG, Jetten MSM (1998). The sequencing batch reactor as a powerful tool for the study of slowly growing anaerobic ammonium-oxidizing microorganisms. Appl Microbiol Biotechnol.

[22] Chu ZR, Wang K, Li XK, Zhu MT, Yang L, Zhang J (2015). Microbial characterization of aggregates within a one-stage nitritation-anammox system using high-throughput amplicon sequencing. Chemica En J.

[23] Kindaichi T, Ito T, Okabe S (2004). Ecophysiological Interaction between Nitrifying Bacteria and Heterotrophic Bacteria in Autotrophic Nitrifying Biofilms as Determined by Microautoradiography-Fluorescence In Situ Hybridization. Appl Environ Microbiol.

[24] Strous M, Pelletier E, Mangenot S, Rattei T, Lehner A, Taylor MW (2006). Deciphering the evolution and metabolism of an anammox bacterium from a community genome. Nature.

[25] Cho S, Fujii N, Lee T, Okabe S (2011). Development of a simultaneous partial nitrification and anaerobic ammonia oxidation process in a single reactor. Bioresour Technol.

[26] Lawson CE, Wu S, Bhattacharjee AS, Hamilton JJ, McMahon KD, Goel R (2017). Metabolic network analysis reveals microbial community interactions in anammox granules. Nat Commun.

[27] Zhao Y, Liu SS, Jiang B, Feng Y, Zhu T, Tao H (2018). Genome-Centered Metagenomics Analysis Reveals the Symbiotic Organisms Possessing Ability to Cross-Feed with Anammox Bacteria in Anammox Consortia. Environ Sci Technol.

[28] Gao D, Liu L, Liang H, Wu WM (2011). Aerobic granular sludge: Characterization, mechanism of granulation and application to wastewater treatment. Critical Rev Biotechnol.

[29] Xia Y, Wang Y, Wang Y, Chin FYL, Zhang T (2016). Cellular adhesiveness and cellulolytic capacity in Anaerolineae revealed by omics-based genome interpretation. Biotechnol Biofuels.

[30] Sekiguchi Y, Takahashi H, Kamagata Y, Ohashi A, Harada H (2001). In Situ Detection, Isolation, and Physiological Properties of a Thin Filamentous Microorganism Abundant in Methanogenic Granular Sludges: A Novel Isolate Affiliated with a Clone Cluster, the Green Non-Sulfur Bacteria. Subdivision I Appl Environ Microbiol.

[31] Björnsson L, Hugenholtz P, Tyson GW, Blackall LL (2002). Filamentous Chloroflexi (green non-sulfur bacteria) are abundant in wastewater treatment processes with biological nutrient removal. Microbiology (N Y).

[32] Li J, Hu B, Zheng P, Qaisar M, Mei L (2008). Filamentous granular sludge bulking in a laboratory scale UASB reactor. Bioresour Technol.

[33] Song YX, Liao Q, Yu C, Xiao R, Tang CJ, Chai LY (2017). Physicochemical and microbial properties of settled and floating anammox granules in upflow reactor. Biochem Eng J.

[34] Nierychlo M, McIlroy SJ, Kucheryavskiy S, Jiang C, Ziegler AS, Kondrotaite Z (2020). Candidatus Amarolinea and Candidatus Microthrix Are Mainly Responsible for Filamentous Bulking in Danish Municipal Wastewater Treatment Plants. Front Microbiol.

[35] Borzacconi L, López I, Passeggi M, Etchebehere C, Barcia R (2008). Sludge deterioration in a full scale UASB reactor after a pH drop working under low loading conditions. Water Sci Technol.

[36] Bovio P, Cabezas A, Etchebehere C (2019). Preliminary analysis of Chloroflexi populations in full-scale UASB methanogenic reactors. J Appl Microbiol.

[37] Juan-Díaz X, Pérez J, Carrera J (2021). Effective dampening of temperature effects in an anammox reactor treating real mainstream wastewater. J Water Proc Eng.

[38] Isanta E, Bezerra T, Fernández I, Suárez-Ojeda ME, Pérez J, Carrera J (2015). Microbial community shifts on an anammox reactor after a temperature shock using 454-pyrosequencing analysis. Bioresour Technol.

[39] Albertsen M, Hugenholtz P, Skarshewski A, Nielsen KL, Tyson GW, Nielsen PH (2013). Genome sequences of rare, uncultured bacteria obtained by differential coverage binning of multiple metagenomes. Nat Biotechnol.

[40] Amann RI, Ludwig W, Schleifer KH (1995). Phylogenetic identification and in situ detection of individual microbial cells without cultivation. Microbiol Rev.

[41] Claesson MJ, O’Sullivan O, Wang Q, Nikkilä J, Marchesi JR, Smidt H (2009). Comparative analysis of pyrosequencing and a phylogenetic microarray for exploring microbial community structures in the human distal intestine. PLoS One.

[42] Callejas C, Fernández A, Passeggi M, Wenzel J, Bovio P, Borzacconi L (2019). Microbiota adaptation after an alkaline pH perturbation in a full-scale UASB anaerobic reactor treating dairy wastewater. Bioprocess Biosyst Eng.

[43] Bolyen E, Rideout JR, Caporaso JG (2019). Reproducible, interactive, scalable and extensible microbiome data science using QIIME 2. Nat Biotechnol.

[44] Nierychlo M, Andersen KS, Xu Y, Green N, Jiang C, Albertsen M (2020). MiDAS 3: An ecosystem-specific reference database, taxonomy and knowledge platform for activated sludge and anaerobic digesters reveals species-level microbiome composition of activated sludge. Water Res.

[45] Andrews. FastQC: a quality control tool for high throughput sequence data. Babraham Institute. 2010. Available from: https://www.bioinformatics.babraham.ac.uk/projects/fastqc/

[46] Bolger AM, Lohse M, Usadel B (2014). Trimmomatic: a flexible trimmer for Illumina sequence data. Bioinformatics.

[47] Li D, Luo R, Liu CM, Leung CM, Ting HF, Sadakane K (2016). MEGAHIT v10: A fast and scalable metagenome assembler driven by advanced methodologies and community practices. Methods.

[48] Kim D, Langmead B, Salzberg SL (2015). HISAT: A fast spliced aligner with low memory requirements. Nat Methods.

[49] Kang DD, Froula J, Egan R, Wang Z (2015). MetaBAT, an efficient tool for accurately reconstructing single genomes from complex microbial communities. PeerJ.

[50] Parks DH, Imelfort M, Skennerton CT, Hugenholtz P, Tyson GW (2015). CheckM: Assessing the quality of microbial genomes recovered from isolates, single cells, and metagenomes. Genome Res.

[51] Parks DH, Chuvochina M, Waite DW, Rinke C, Skarshewski A, Chaumeil PA (2018). A standardized bacterial taxonomy based on genome phylogeny substantially revises the tree of life. Nat Biotechnol.

[52] Bankevich A, Nurk S, Antipov D, Gurevich AA, Dvorkin M, Kulikov AS (2012). SPAdes: A new genome assembly algorithm and its applications to single-cell sequencing. J Comput Biol.

[53] Ultsch A, Fabian M, Mörchen F. ESOM-Maps: tools for clustering, visualization, and classification with Emergent SOM. Univ., 2005. Vol. 46.

[54] Orakov A, Fullam A, Coelho LP, Khedkar S, Szklarczyk D, Mende DR (2021). GUNC: detection of chimerism and contamination in prokaryotic genomes. Genome Biol.

[55] Jain C, Rodriguez-R LM, Phillippy AM, Konstantinidis KT, Aluru S (2018). High throughput ANI analysis of 90K prokaryotic genomes reveals clear species boundaries. Nature Commun.

[56] Kim D, Park S, Chun J (2021). Introducing EzAAI: a pipeline for high throughput calculations of prokaryotic average amino acid identity. J Microbiol.

[57] Letunic I, Bork P (2019). Interactive Tree of Life (iTOL) v4: Recent updates and new developments. Nucleic Acids Res.

[58] Hyatt D, Chen GL, LoCascio PF, Land ML, Larimer FW, Hauser LJ (2010). Prodigal: Prokaryotic gene recognition and translation initiation site identification. BMC Bioinformatics.

[59] Seemann T. Prokka: rapid prokaryotic genome annotation. Bioinforma. 2014;30(14):2068-9. 10.1093/bioinformatics/btu153 24642063

[60] Kanehisa M, Goto S (2000). KEGG: Kyoto Encyclopedia of Genes and Genomes [Internet]. Nucleic Acids Research.

[61] Dong X, Strous M (2019). An Integrated Pipeline for Annotation and Visualization of Metagenomic Contigs. Front Genet.

[62] Cantarel BL, Coutinho PM, Rancurel C, Bernard T, Lombard V, Henrissat B. The Carbohydrate-Active EnZymes database (CAZy): an expert resource for glycogenomics. Nucleic Acids Res. 2009;37(suppl_1):D233-8. 10.1093/nar/gkn663 PMC2686590 18838391

[63] Nierychlo M, Andersen KS, Xu Y, Green N, Jiang C, Albertsen M, Dueholm MS, Nielsen PH. MiDAS 3: an ecosystem-specific reference database, taxonomy and knowledge platform for activated sludge and anaerobic digesters reveals species-level microbiome composition of activated sludge. Water Res. 2020;182:115955. 10.1016/j.watres.2020.115955 32777640

[64] Mielczarek AT, Kragelund C, Eriksen PS, Nielsen PH (2012). Population dynamics of filamentous bacteria in Danish wastewater treatment plants with nutrient removal. Water Res.

[65] Gonzalez-Gil G, Sougrat R, Behzad AR, Lens PNL, Saikaly PE (2015). Microbial Community Composition and Ultrastructure of Granules from a Full-Scale Anammox Reactor. Microb Ecol.

[66] Pereira AD, Cabezas A, Etchebehere C, de Chernicharo CAL, de Araújo JC (2017). Microbial communities in anammox reactors: a review. Environ Technol Rev.

[67] Campanaro S, Treu L, Rodriguez-R LM, Kovalovszki A, Ziels RM, Maus I (2020). New insights from the biogas microbiome by comprehensive genome-resolved metagenomics of nearly 1600 species originating from multiple anaerobic digesters. Biotechnol Biofuels.

[68] Speth DR, in’t Zandt MH, Guerrero-Cruz S, Dutilh BE, Jetten MS. Genome-based microbial ecology of anammox granules in a full-scale wastewater treatment system. Nat Commun. 2016;7(1):11172. 10.1038/ncomms11172 PMC4821891 27029554

[69] Nierychlo M, Miłobȩdzk A, Petriglieri F, McIlroy B, Nielsen PH, McIlroy SJ (2019). The morphology and metabolic potential of the Chloroflexi in full-scale activated sludge wastewater treatment plants. FEMS Microbiol Ecol.

[70] Bowers RM, Kyrpides NC, Stepanauskas R, Harmon-Smith M, Doud D, Reddy TBK (2017). Minimum information about a single amplified genome (MISAG) and a metagenome-assembled genome (MIMAG) of bacteria and archaea [Internet]. Nature Biotechnology.

[71] Parks DH, Chuvochina M, Chaumeil PA, Rinke C, Mussig AJ, Hugenholtz P (2020). A complete domain-to-species taxonomy for Bacteria and Archaea. Nature Biotechnol.

[72] Blazejak A, Schippers A (2010). High abundance of JS-1- and Chloroflexi-related Bacteria in deeply buried marine sediments revealed by quantitative, real-time PCR. FEMS Microbiol Ecol.

[73] Rosselló-Móra R, Amann R (2015). Past and future species definitions for Bacteria and Archaea. Syst Appl Microbiol.

[74] Konstantinidis KT, Rosselló-Móra R, Amann R (2017). Uncultivated microbes in need of their own taxonomy. ISME Journal.

[75] Yarza P, Yilmaz P, Pruesse E, Glöckner FO, Ludwig W, Schleifer KH (2014). Uniting the classification of cultured and uncultured bacteria and archaea using 16S rRNA gene sequences. Nat Rev Microbiol.

[76] Xia Y, Kong Y, Nielsen PH (2007). In situ detection of protein-hydrolysing microorganisms in activated sludge. FEMS Microbiol Ecol.

[77] Okabe S, Kindaichi T, Ito T (2005). Fate of 14C-labeled microbial products derived from nitrifying bacteria in autotrophic nitrifying biofilms. Appl Environ Microbiol.

[78] Zang K, Kurisu F, Kasuga I, Furumai H, Yagi O (2008). Analysis of the phylogenetic diversity of estrone-degrading bacteria in activated sewage sludge using microautoradiography-fluorescence in situ hybridization. Syst Appl Microbiol.

[79] Miura Y, Okabe S (2008). Quantification of cell specific uptake activity of microbial products by uncultured chloroflexi by microautoradiography combined with fluorescence in situ hybridization. Environ Sci Technol.

[80] Kragelund C, Thomsen TR, Mielczarek AT, Nielsen PH (2011). Eikelboom’s morphotype 0803 in activated sludge belongs to the genus Caldilinea in the phylum Chloroflexi. FEMS Microbiol Ecol.

[81] Campbell AG, Schwientek P, Vishnivetskaya T, Woyke T, Levy S, Beall CJ (2014). Diversity and genomic insights into the uncultured Chloroflexi from the human microbiota. Environ Microbiol.

[82] McGonigle JM, Lang SQ, Brazelton WJ (2019). Genomic Evidence for Formate Metabolism by Chloroflexi as the Key to Unlocking Deep Carbon in Lost City Microbial Ecosystems. Appl Environ Microbiol.

[83] Hug LA, Castelle CJ, Wrighton KC, Thomas BC, Sharon I, Frischkorn KR (2013). Community genomic analyses constrain the distribution of metabolic traits across the Chloroflexi phylum and indicate roles in sediment carbon cycling. Microbiome.

[84] Wasmund K, Schreiber L, Lloyd KG, Petersen DG, Schramm A, Stepanauskas R (2014). Genome sequencing of a single cell of the widely distributed marine subsurface Dehalococcoidia, phylum Chloroflexi. ISME J.

[85] Heider J, Ma K, Adams MWW (1995). Purification, characterization, and metabolic function of tungsten- containing aldehyde ferredoxin oxidoreductase from the hyperthermophilic and proteolytic archaeon Thermococcus strain ES-1. J Bacteriol.

[86] Ali M, Shaw DR, Albertsen M, Saikaly PE (2020). Comparative Genome-Centric Analysis of Freshwater and Marine ANAMMOX Cultures Suggests Functional Redundancy in Nitrogen Removal Processes. Front Microbiol.

[87] Zhao Y, Jiang B, Tang X, Liu S (2019). Metagenomic insights into functional traits variation and coupling effects on the anammox community during reactor start-up. Sci Total Environ.

[88] Narihiro T, Terada T, Ohashi A, Kamagata Y, Nakamura K, Sekiguchi Y (2012). Quantitative detection of previously characterized syntrophic bacteria in anaerobic wastewater treatment systems by sequence-specific rRNA cleavage method. Water Res.

[89] Singleton C, Petriglieri F, Kristensen J, Kirkegaard R, Michaelsen T, Andersen M (2020). Connecting structure to function with the recovery of over 1000 high-quality activated sludge metagenome-assembled genomes encoding full-length rRNA genes using long-read sequencing. Nat Commun.

[90] McIlroy SJ, Onetto CA, McIlroy B, Herbst FA, Dueholm MS, Kirkegaard RH (2018). Genomic and in Situ Analyses reveal the Micropruina spp as Abundant fermentative glycogen accumulating organisms in enhanced biological phosphorus removal systems. Front Microbiol.

[91] Lee SY. Bacterial polyhydroxyalkanoates. Biotechnology and bioengineering. 1996;49(1):1-14. 10.1002/(SICI)1097-0290(19960105)49:1<1::AID-BIT1>3.0.CO;2-P 18623547

[92] Schlegel HG, Gottschalk G, Von Bartha R (1961). Formation and utilization of poly-β-hydroxybutyric acid by knallgas bacteria (hydrogenomonas). Nature.

[93] Kalscheuer R, Steinbüchel A (2003). A novel bifunctional wax ester synthase/acyl-CoA: Diacylglycerol acyltransferase mediates wax ester and triacylglycerol biosynthesis in Acinetobacter calcoaceticus ADP1. J Biol Chem.

[94] Kawaichi S, Yoshida T, Sako Y, Nakamuraa R, Nakamura R (2015). Draft genome sequence of a heterotrophic facultative anaerobic thermophilic bacterium, Ardenticatena maritima strain 110ST. Genome Announc.

[95] Lawson CE, Wu S, Bhattacharjee AS, Hamilton JJ, McMahon KD, Goel R (2017). Metabolic network analysis reveals microbial community interactions in anammox granules. Nat Commun.

[96] Watterson WJ, Tanyeri M, Watson AR, Cham CM, Shan Y, Chang EB (2020). Droplet-based high-throughput cultivation for accurate screening of antibiotic resistant gut microbes. Elife.

