# Supplementary material for: Genome-centric metagenomic insights into the role of Chloroflexi in anammox, activated sludge and methanogenic reactors
Source: BMC Microbiol. 2023 Feb 21;23:45. doi: 10.1186/s12866-023-02765-5 (PMC9942424; doi:10.1186/s12866-023-02765-5)
Supplement: Supplementary file 1 — Additional file 1. [file 12866_2023_2765_MOESM1_ESM.docx]

**Supplementary information:**

Genome-centric metagenomic insights into the role of Chloroflexi in anammox, activated sludge and methanogenic reactors

Patricia Bovio-Winkler, Leandro D. Guerrero, Leonardo Erijman, Pía Oyarzúa, María Eugenia Suárez Ojeda, Angela Cabezas and Claudia Etchebehere

**Materials and methods**

Fluorescence *in situ* hybridization (FISH). FISH analysis was performed to determine the morphology of members affiliated to the Chloroflexi using the specifics probes GNSB941 and CFX1223R labeled with Cy3 (1,2). The hybridization was performed according to Björnsson et al. (1). For cellular staining 4’, 6’ -diamin-2-phenylindole (DAPI) was used. The pure culture of *Sphaerobacter thermophilus* strain DSM 20745 was used as positive control, and *Escherichia coli* strain DH5α was used as negative control. The glass slides with the samples were embedded in Citifluor AF1 (Citifluor, Canterbury, United Kingdom). Images of fluorescent cells were recorded using a confocal laser scanning microscope (LSM 800, Carl Zeiss, Oberkochen, Germany).

**DNA extraction**

For DNA extraction, sludge samples were thawed and centrifuged (5 min, 10,000 g). Approximately 0.35 g of wet pellet was used for DNA extraction with the ZR Soil Microbe DNA MiniPrepTM kit (Zymo Research) according to the manufacturer’s instructions. The quality of the extracted genomic DNA was determined by 1% agarose gel electrophoresis (Nucleic Acid Stain, GoodViewTM, Beijing) and stored at -20 °C until further use.

**Amplicon sequencing analyses and visualization**

Samples were sequenced on an Ion Torrent PGM (Life Technologies) in the platform at the Biological Research Institute ‘Clemente Estable’ (Montevideo, Uruguay). Multiplexed single-end sequencing reads (1,628,931 in total) were imported into the QIIME2. The ‘divisive amplicon denoising algorithm’ DADA2 (3) plugin in QIIME2 was used to ‘denoise’ sequencing reads. This step filters out noise and corrects errors in marginal sequences, removes chimeric sequences and singletons and finally dereplicates the resulting sequences, resulting in high resolution amplicon sequence variants (ASVs) for downstream analysis. The consensus sequences for the ASVs were classified with a classify-sklearn classifier trained against the pre-trained classifier MiDAS 3.7 database (4). The sequences were submitted to the NCBI (http://ncbi.nlm.nih.gov) under accession BioProject ID PRJNA780299. All the statistical analysis was performed in R version 3.5.1 (R Core Team 2020) with R Studio environment Version 1.3.1093. The biom file from QIIME2 was imported and analyzed through phyloseq-modified workflow (5). Sequences were rarefied to the lowest number of sequences per sample (n= 40,016 sequences). In order to determine the taxonomic composition of Chloroflexi in each sample, a specific analysis of the sequencing data was performed using only the sequences affiliated to the phylum Chloroflexi. We calculated the relative abundance of the phylum Chloroflexi at different taxonomic levels using the total of sequences of Chloroflexi in each sample (normalization). Barplots and heatmaps were generated using ampvis2 (v.2.6.5) (6).

**Phylogenetic analysis of the amplicon sequences of the phylum Chloroflexi**

A phylogenetic tree was constructed to evaluate the phylogenetic position of the ASVs classified within the phylum Chloroflexi. It included ASVs with a relative abundance greater than 5% in at least one sample, the 16S rRNA sequences from assembled genomes (retrieved from the metagenomes as will be explained) and sequences from cultured representatives and MAGs retrieved from the database (NCBI). Four 16S rRNA gene sequences from different genera within the phylum Thermotogota served as an outgroup for rooting the tree. The phylogenetic tree was performed using the maximum likelihood method with the generalized time reversible (GTR) (7) substitution model and GAMMA distribution model using 1,000 bootstraps in MEGA 10.0 (8). The resultant phylogenetic tree was visualized in iTOL (9).

**Results and discussion**

**Morphology determined by FISH**

The filamentous morphology of Chloroflexi is an important characteristic as it might be involved in floc and granule formation. We performed FISH using specific probes for the phylum Chloroflexi. Different filamentous morphology such as thin and thick filaments (with or without septum) were observed in samples from reactors RH and MO (Fig. S1a and b). In samples taken from the anammox reactor, the signal was very low and filamentous morphology was not observed (Fig. S1c). Instead, hybridization with non-filamentous cells was observed. Interference of polysaccharides in the FISH technique in anammox granules from the same reactor was previously reported (10). Thus, the presence of filamentous morphology in the anammox samples must be further investigated.


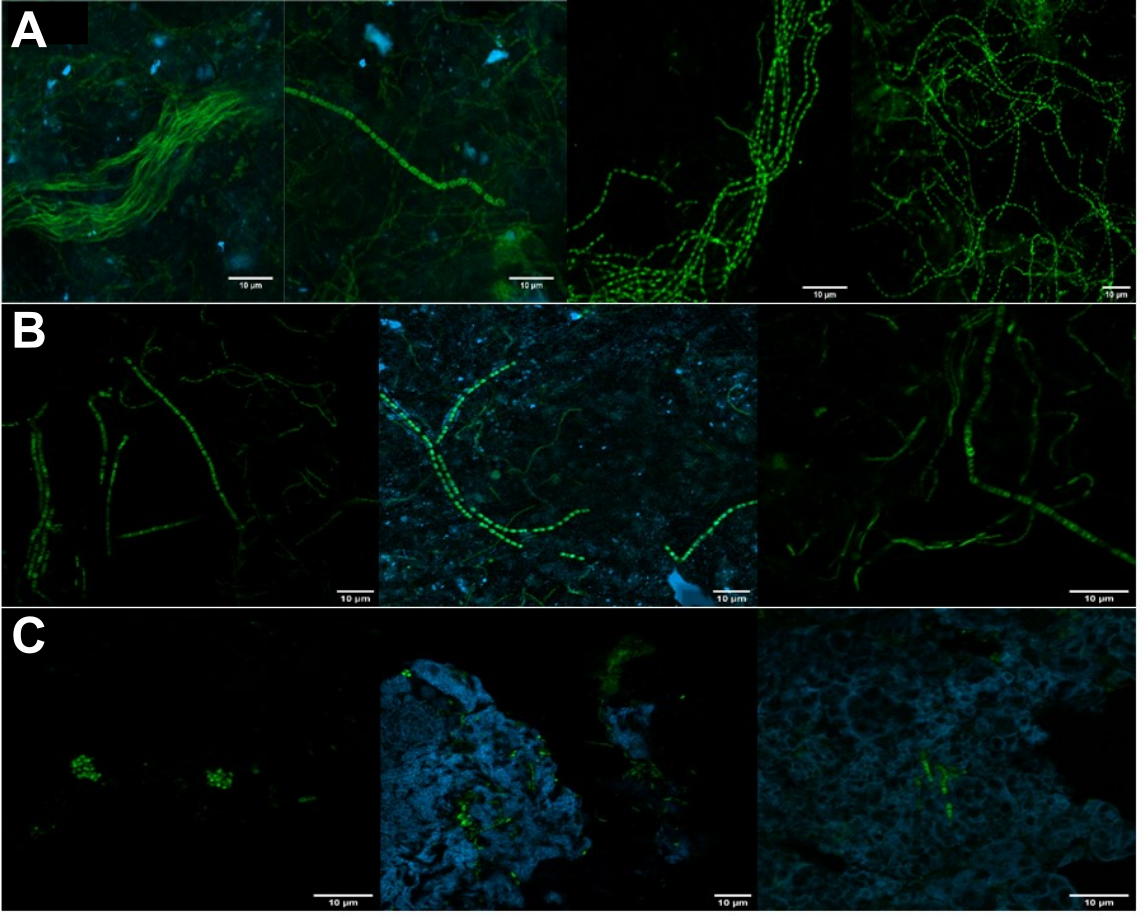


Figure S1 FISH micrographs taken from samples from RH (A), MO (B) and UAnSB (S) (C) reactors by confocal laser scanning microscopy (digitally produced images with pseudo-colors). The samples were simultaneously hybridized with the probes GNSB941 and CFX1223R Cy5-labelled (green) directed to the phylum Chloroflexi, and DAPI staining (blue) (Scale bar, 10 µm)


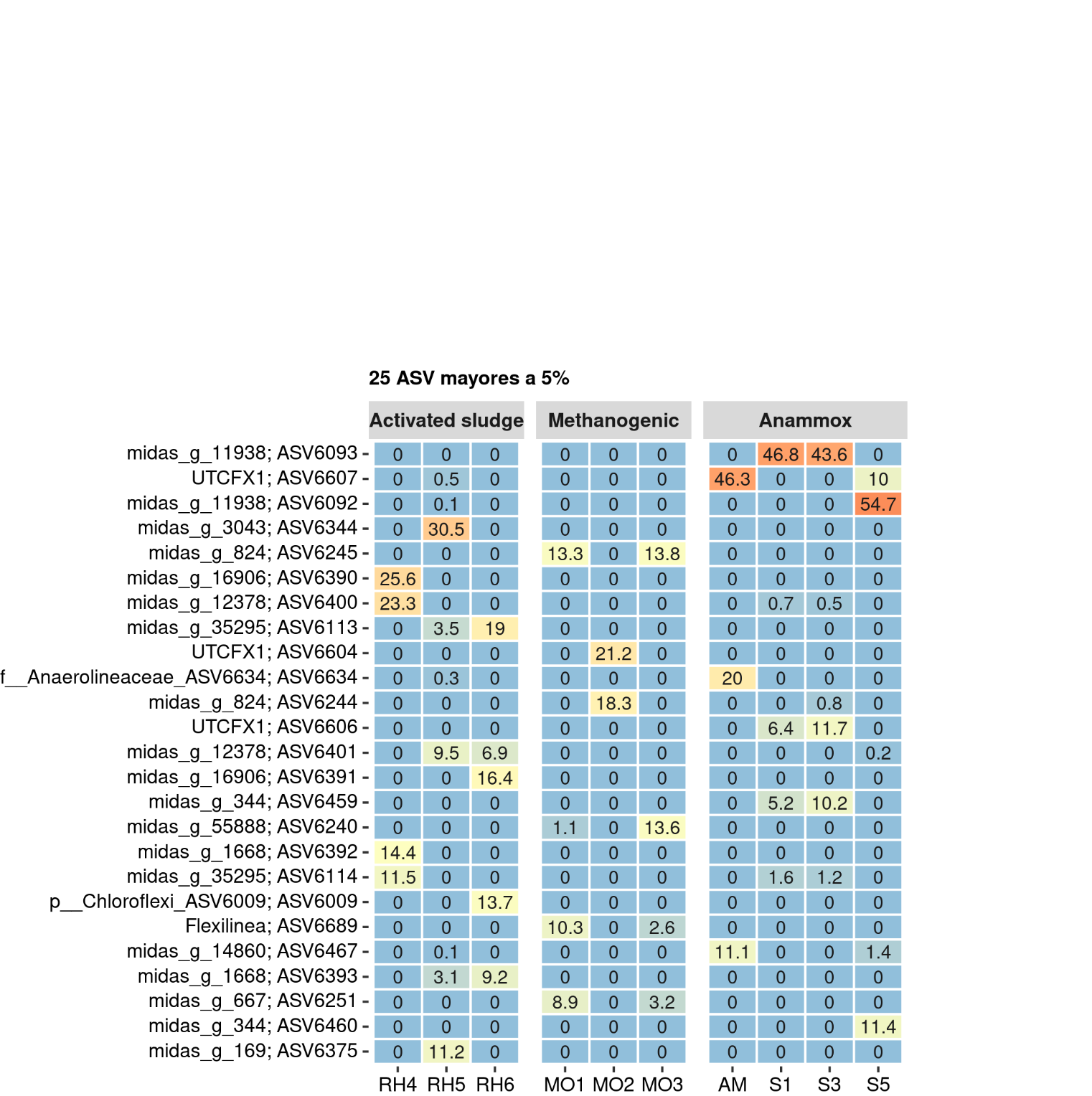


Figure S2 Taxonomic composition of the Chloroflexi community in the samples taken from the three reactors according to the 16S rRNA gene amplicon sequence at ASV level

Table S1 ANI values between MAGs and between reference genomes. The highest ANI values obtained between genomes are shown

| Chloroflexi MAGs | Most related MAG or isolated or Candidates species | ANI (%) | AAI (%) | Genus | Species |
| --- | --- | --- | --- | --- | --- |
| RH21 | AMX9 | 75.5 | 57.6 | ≠ | ≠ |
| RH38 | AMX56 | 76.0 | 56.3 | ≠ | ≠ |
| RH43 | RH38 | 75.6 | 62.4 | ≠ | ≠ |
| RH52 | RH38 | 75.7 | 53.1 | ≠ | ≠ |
| MO16 | MO118 | 77.4 | 56.1 | ≠ | ≠ |
| MO53 | MO118 | 75.9 | 51.0 | ≠ | ≠ |
| MO66 | AMX56 | 75.8 | 56.0 | ≠ | ≠ |
| MO118 | AMX56 | 78.7 | 50.9 | ≠ | ≠ |
| AMX9 | MO118 | 76.7 | 51.0 | ≠ | ≠ |
| AMX14 | AMX68 | 79.7 | 78.2 | = | ≠ |
| AMX15 | MO118 | 79.6 | 54.3 | ≠ | ≠ |
| AMX39 | AMX15 | 79.6 | 78.3 | = | ≠ |
| AMX47 | RH38 | 75.5 | 50.4 | ≠ | ≠ |
| AMX55 | AMX15 | 77.7 | 66.8 | = | ≠ |
| AMX55 | AMX56 | 75.5 | 53.4 | ≠ | ≠ |
| AMX56 | MO118 | 76.5 | 51.0 | ≠ | ≠ |
| AMX57 | AMX9 | 75.7 | 58.9 | ≠ | ≠ |
| RH21 | Flexilinea flocculi GCA_001192795.1 | 76.7 | 50.7 | ≠ | ≠ |
| RH38 | Caldilinea aerophila GCF_000281175.1 | 77.3 | 68.8 | = | ≠ |
| RH43 | Litorilinea aerophila GCF_006569185.1 | 75.9 | 65.4 | = | ≠ |
| RH52 | Leptolinea tardivitalis GCF_001306095.1 | 77.9 | 51.6 | ≠ | ≠ |
| MO16 | Longilinea arvoryzae GCF_001050235.1 | 79.4 | 77.2 | = | ≠ |
| MO53 | *Ca*. Roseilinea Naka GCF_012931985.1 | 75.2 | 52.3 | ≠ | ≠ |
| MO66 | Amarolinea aalborgensis GCA_900491745.1 | 76.5 | 58.8 | ≠ | ≠ |
| MO118 | Flexilinea flocculi GCF_001192795.1 | 83.2 | 87.4 | = | ≠ |
| AMX55 | Leptolinea tardivitalis GCF_001306095.1 | 76.3 | 57.6 | ≠ | ≠ |
| AMX68 | Flexilinea flocculi GCF_001192795.1 | 75.8 | 54.3 | ≠ | ≠ |
| AMX14 | Flexilinea flocculi GCF_001192795.1 | 76.1 | 54.2 | ≠ | ≠ |
| AMX39 | Leptolinea tardivitalis GCF_001050275.1 | 76.3 | 57.0 | ≠ | ≠ |
| AMX15 | Flexilinea flocculi GCF_001192795.1 | 80.5 | 54.2 | ≠ | ≠ |
| AMX9 | Flexilinea flocculi GCF_001192795.1 | 75.9 | 51.1 | ≠ | ≠ |
| AMX57 | Aggregatilinea lenta GCF_003569045.1 | 75.7 | 57.9 | ≠ | ≠ |
| AMX56 | Amarolinea aalborgensis GCA_900491745.1 | 75.8 | 56.6 | ≠ | ≠ |
| AMX47 | Tepidiforma bonchosmolovskayae GCF_008838325.1 | 77.6 | 63.1 | ≠ | ≠ |

ANI: average nucleotide identity

AAI: average amino acid identity

Table S2. Taxonomic classification of the genomes of the proposed new Candidates (GTDB-tk database) and their 16S rRNA gene sequences (MiDAS and SILVA databases)

| MAG |  | Database | Taxonomy | Identity (%) |
| --- | --- | --- | --- | --- |
| AMX47 | genome | GTDB-tk 95 | c_Dehalococcoidia;o_UBA2991;f_UBA2991;g_UCB2 | 82.41 |
|  | 16S rRNA gene (NODE_202) | SILVA 138.1 | Unclassified | 91.35 |
|  |  | MIDAS 4.8.1 | c_OLB14;midas_o_731;f_midas_f_731;g_midas_g_1412;midas_s_8871 | 94.66 |
| AMX55 | genome | GTDB-tk 95 | c_Anaerolineae;o_Anaerolineales;f_EnvOPS12;g_UBA7227;s_UBA7227 sp002473085 | 98.72 |
|  | AMX55 16S rRNA gene (NODE_28) | SILVA 138.1 | c_Anaerolineae;o_Anaerolineales;f_Anaerolineaceae;g_UTCFX1 | 96.99 |
|  |  | MIDAS 4.8.1 | c_Anaerolineae;o_Anaerolineales;f_Anaerolineaceae;g_Ca Villigracilis;midas_s_3088 | 95.68 |
| AMX56 | genome | GTDB-tk 95 | c_Anaerolineae;o_Caldilineales;f_J102 | 76.67 |
|  | AMX56 16S rRNA gene (NODE_14) | SILVA 138.1 | Unclassified | 91.39 |
|  |  | MIDAS 4.8.1 | c_Anaerolineae;o_midas_o_1;f_midas_f_813;midas_g_7034;midas_s_7034 | 92.95 |
|  | AMX56 16S rRNA gene (NODE_94) | SILVA 138.1 | c_Anaerolineae;o_Anaerolineales;f_Anaerolineaceae | hole98.15 |
|  |  | MIDAS 4.8.1 | c_Anaerolineae;o_Anaerolineales;f_Anaerolineaceae;g_Ca_Villigracilis;midas_s_3088 | 96.41 |


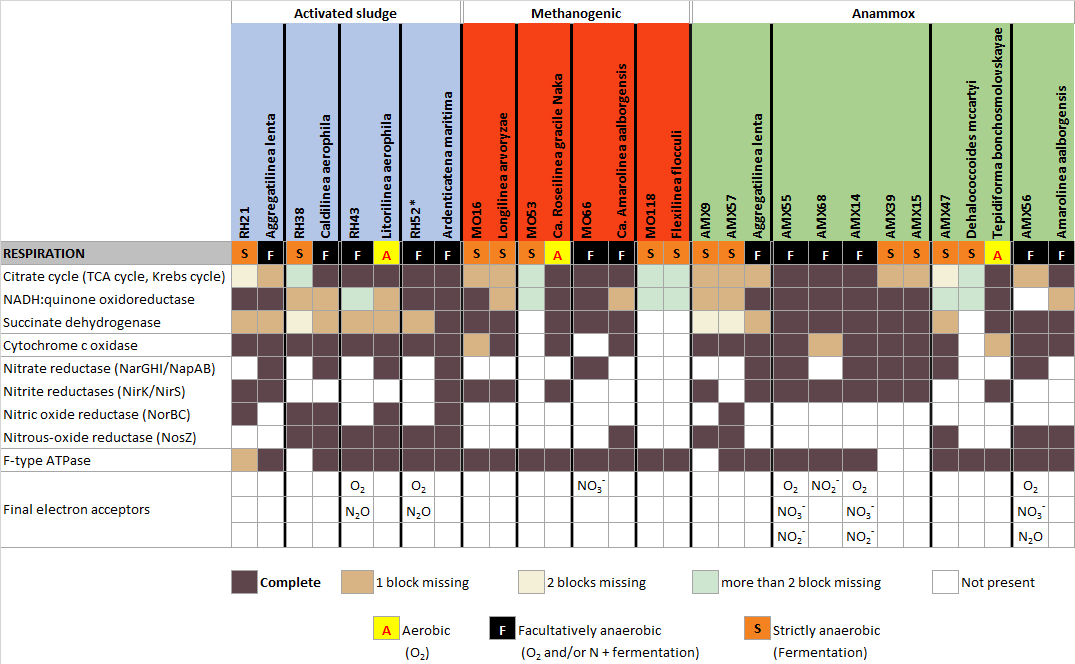


Figure S3 Heatmap showing the completeness and incompleteness of the metabolic pathways in each MAG of Chloroflexi and in genomes of isolated species of Chloroflexi (and also Candidatus) closely related to the MAGs. We considered that species could use the O2 as final electron acceptor if were present the following enzymes or modules: TCA cycle, NADH:quinone oxidoreductase, succinate dehydrogenase, cytochrome c oxidase and F-type ATPase.

1. Björnsson L, Hugenholtz P, Tyson GW, Blackall LL. Filamentous Chloroflexi (green non-sulfur bacteria) are abundant in wastewater treatment processes with biological nutrient removal. Microbiology. 2002;148(8):2309–18.

2. Gich F, Garcia-Gil J, Overmann J. Previously unknown and phylogenetically diverse members of the green nonsulfur bacteria are indigenous to freshwater lakes. Arch Microbiol. 2002;177(1):1–10.

3. Callahan BJ, McMurdie PJ, Rosen MJ, Han AW, Johnson AJA, Holmes SP. DADA2: High-resolution sample inference from Illumina amplicon data. Nat Methods [Internet]. 2016 Jul 23 [cited 2019 Apr 5];13(7):581–3. Available from: http://www.nature.com/articles/nmeth.3869

4. Nierychlo M, Andersen KS, Xu Y, Green N, Jiang C, Albertsen M, et al. MiDAS 3: An ecosystem-specific reference database, taxonomy and knowledge platform for activated sludge and anaerobic digesters reveals species-level microbiome composition of activated sludge. Water Res [Internet]. 2020;115955. Available from: https://doi.org/10.1016/j.watres.2020.115955

5. McMurdie PJ, Holmes S. phyloseq: An R Package for Reproducible Interactive Analysis and Graphics of Microbiome Census Data. Watson M, editor. PLoS One [Internet]. 2013 Apr 22 [cited 2021 Jan 18];8(4):e61217. Available from: https://dx.plos.org/10.1371/journal.pone.0061217

6. Andersen KS, Kirkegaard RH, Karst SM, Albertsen M. ampvis2: An R package to analyse and visualise 16S rRNA amplicon data [Internet]. bioRxiv. bioRxiv; 2018 [cited 2021 Jan 18]. p. 299537. Available from: https://doi.org/10.1101/299537

7. Nei M, Kumar S. Molecular evolution and phylogenetics [Internet]. Oxford University Press; 2000 [cited 2019 Mar 21]. 333 p. Available from: https://global.oup.com/academic/product/molecular-evolution-and-phylogenetics-9780195135855?cc=uy&lang=en&

8. Kumar S, Stecher G, Li M, Knyaz C, Tamura K. MEGA X: Molecular Evolutionary Genetics Analysis across Computing Platforms. Battistuzzi FU, editor. Mol Biol Evol [Internet]. 2018 Jun 1 [cited 2019 Nov 28];35(6):1547–9. Available from: https://academic.oup.com/mbe/article/35/6/1547/4990887

9. Letunic I, Bork P. Interactive Tree of Life (iTOL) v4: Recent updates and new developments. Nucleic Acids Res. 2019;47(W1):256–9.

10. Oyarzúa P, Bovio-Winkler P, Etchebehere C, Suárez-Ojeda ME. Microbial communities in an anammox reactor treating municipal wastewater at mainstream conditions: Practical implications of different molecular approaches. J Environ Chem Eng. 2021;9(6):106622.
